# The efficacy of Life Review Therapy combined with Memory Specificity Training (LRT-MST) targeting cancer patients in palliative care: A randomized controlled trial

**DOI:** 10.1371/journal.pone.0197277

**Published:** 2018-05-15

**Authors:** Gitta Kleijn, Birgit I. Lissenberg-Witte, Ernst T. Bohlmeijer, Bas Steunenberg, Kitty Knipscheer-Kuijpers, Vincent Willemsen, Annemarie Becker, Egbert F. Smit, Corien M. Eeltink, Anna M. E. Bruynzeel, Maurice van der Vorst, Remco de Bree, C. René Leemans, Michiel W. M. van den Brekel, Pim Cuijpers, Irma M. Verdonck-de Leeuw

**Affiliations:** 1 Department of Clinical, Neuro- & Developmental Psychology, Vrije Universiteit Amsterdam, Amsterdam, The Netherlands; 2 Department of Epidemiology and Biostatistics, VU University Medical Center, Amsterdam, The Netherlands; 3 Department of Mental Health, University Twente, Enschede, The Netherlands; 4 University of Applied Sciences Utrecht, Faculty Health Sciences, Utrecht, the Netherlands; 5 Center for Psychosocial Oncology Care, Ingeborg Douwes Center, Amsterdam, The Netherlands; 6 Department of Pulmonary Diseases, VU University Medical Center, Amsterdam, The Netherlands; 7 Department of Haematology, VU University Medical Center, Amsterdam, The Netherlands; 8 Department of Radiation Oncology, VU University Medical Center, Amsterdam, The Netherlands; 9 Department of Medical Oncology, VU University Medical Center, Amsterdam, The Netherlands; 10 Department of Head and Neck Surgery, VU University Medical Center, Amsterdam, The Netherlands; 11 Department of Otolaryngology / Head and Neck Surgery, Netherlands Cancer Institute, Amsterdam, The Netherlands; TNO, NETHERLANDS

## Abstract

**Background:**

The aim of this study was to evaluate the efficacy of an intervention combining Life Review Therapy (LRT) and Memory Specificity Training (MST) (LRT-MST) to improve ego-integrity and despair among cancer patients in palliative care.

**Methods:**

In this multicentre randomized controlled trial, cancer patients in palliative care were randomized to the intervention group (LRT-MST; n = 55) or waiting-list control group (n = 52). LRT-MST is a 4-session home-based psychological intervention that aims to retrieve specific positive memories, to re-evaluate life events and to reconstruct the story of a patient’s life, including the diagnosis of incurable cancer. Outcome measures were ego-integrity and despair (NEIS), psychological distress, anxiety and depression (HADS), quality of life (EORTC QLQ-C15-PAL), and specificity of the autobiographical memory (AMT). NEIS, HADS and EORTC QLQ-C15-PAL were assessed at baseline (T0), 1 month later (post-treatment; T1), and at 1 month follow-up (T2). AMT was assessed at T0 and T1. Linear mixed models (intention to treat) were used to assess group differences in changes over time. Independent samples t-tests were used to assess group differences at T0, T1, and T2, and effect sizes (ES) were calculated at T1 and T2.

**Results:**

The course of ego-integrity (not despair) improved significantly over time (*p* = .007) in the intervention group compared to the waiting-list control group, with moderate, but insignificant, effect sizes at T1 (ES = .42) and T2 (ES = .48). Compliance rate was 69% and total dropout rate was 28%, both primarily related to disease progression and death.

**Conclusions:**

LRT-MST seems effective among cancer patients in palliative care to improve the course of ego-integrity.

## Introduction

Being diagnosed with advanced cancer has a great impact on quality of life and studies suggest that psychological, spiritual, and existential issues may be of greater concern to advanced cancer patients than pain and other physical symptoms [1,2]. In 2009, Holland et al. [3] reviewed interventions that may help cancer patients, such as meaning-centered therapy and dignity therapy. Since then, several randomized controlled trials showed evidence of the effectiveness of meaning-centered therapy [4,5] and dignity therapy [6–9] targeting advanced cancer patients. Holland et al. [3] also presented a theoretical framework for the development of new interventions targeting elderly cancer patients utilizing psychological and educational approaches in conjunction with recall of memories from the past (life review).

Life review is defined as ‘the progressive return to consciousness of prior experience, which can be re-evaluated with the intention of resolving and integrating past conflicts, thereby providing new significance to one’s life’ [10]. Reviewing one’s life and coming to terms with their memories may make older persons more accepting and be less anxious about their approaching death [10]. Given that life review is “helping individuals integrate memories into a meaningful whole, and providing a harmonious view of past, present and future” [11], it may lead to greater achievement of ego-integrity. Ego-integrity is described as accepting your life cycle as something that had to be, feeling connected to others, and experiencing a sense of wholeness, meaning and coherence as a person facing (the approach of) death. It is also supposed to be associated with achieving wisdom [3,12–15] and less death anxiety [16]. Achieving ego-integrity is part of Erikson’s eight and final life stage [14]. The absence of achieving ego-integrity is called despair. Despair is described as experiencing regret about one’s life, and associated feelings of sadness, failure and hopelessness. It is also suggested to be related to psychological distress, depressive symptoms, loneliness and isolation [3].

A life review intervention aims to integrate positive and negative life events in a coherent life story. It is a structured variant of reminiscence (recalling memories from the past) and typically addresses distinct lifetime periods such as childhood, adolescence, adulthood, and a life summary including the present time. Life review therapy (LRT) refers to the use of life review in patients with mental health problems [17]. Although life review was developed for elderly [10], not only elderly in the final stage of life are confronted with death, reminiscence, ego-integrity and despair, but people with cancer as well [3]. Pickrel [18] described that a life review process for terminally ill (of any age) can help a person to complete the last chapter of his or her life, to maintain some control, to make things right or finish up unfinished business and to enhance a good feeling at the end. This may result in that one is better able to deal with the loss of life. Among cancer patients, observational studies indicate that LRT decreases depressive feelings and improves spiritual and psychosocial well-being [19,20]. Two randomized controlled trials showed that LRT is indeed effective among terminally ill cancer patients in Japan [21] and China [22]. Although both studies investigated LRT among terminally ill cancer patients, they had different outcome measures, a different number of sessions of LRT and different residences. Both studies showed significant effects on domains related to ego-integrity and despair (life completion [21]; existential distress and value of life [22]), but this was only based on a subscale or domain of a questionnaire. An important aspect in LRT is the autobiographic memory, consisting of memories from an individual's life and knowledge about the world [23,24]. Previous research showed that depression and depressed mood are associated with difficulty in recollecting specific and positive autobiographical memories, which is called reduced memory specificity [24–26]. Reduced memory specificity also predicts increased emotional (depressive) reactivity to stressful events in a non-clinical population and the course of recovery of depressed patients. Memory specificity training has been shown to improve the recall of specific memories in depressed patients [27,28].

Based on these previous studies in palliative cancer patients, of which some experiencing a great sense of end-of-life despair [29] and who are at risk for developing depression [30], we investigated an intervention that combines LRT with memory specificity training (MST) (LRT-MST). This intervention is named ‘Dear Memories’ and focusses on retrieving specific, and positive memories of different lifetime periods and aims to improve autobiographical memory specificity via the structured way of life review therapy.

The aim of the present study was to assess the efficacy of LRT-MST to improve ego-integrity and reduce despair among cancer patients in the palliative phase. Secondary aim was to explore the effect of LRT-MST on psychological distress, anxiety and depression, quality of life, and on the specificity of the autobiographical memory.

## Materials and methods

### Study design and population

This study was a multicentre randomized controlled trial with two parallel groups; an intervention group (receiving LRT-MST) and a waiting-list control group (receiving care-as-usual; CAU). The trial was approved by the Medical Ethics Committee of VU University Medical Center and registered in the Netherlands Trial Register (NTR 2256).

Eligible participants were adult (>18 years old) cancer patients, with all types of cancer and all cancer treatment modalities, receiving palliative care and an expected prognosis of more than 3 months. At the start of this study significant depressive symptoms (HADS (HADS A/D > 7/ HADS-T >14) was an inclusion criteria, but shortly after trial commencement this was not part of the inclusion criteria (and primary outcome) anymore. Exclusion criteria were psychotic behaviour, severe cognitive dysfunction, severe impairment in verbal communication or insufficient mastery of the Dutch language.

Patients who were treated at VU University Medical Center (Departments of Head and Neck Surgery, Pulmonary Diseases, Hematology, Radiotherapy, and Medical Oncology) or the Netherlands Cancer Institute/ Antoni van Leeuwenhoek (Department of Head and Neck Surgery) (after trial commencement the number of departments participating expanded), fulfilled the in- and exclusion criteria, and provided written informed consent, were scheduled for a pre-test assessment session at the patient’s residence, after which they were randomly assigned to either the LRT-MST or CAU group. Clinical characteristics were retrieved from the medical files. The Mini-International Neuropsychiatric Interview (MINI) [31] was used to assess the presence of Major Depressive Disorder (MDD) current and lifetime, according to the criteria of the Diagnostic and Statistical Manual of Mental Disorders IV [32]. The full trial protocol can be requested via the corresponding author.

### Intervention

LRT-MST called ‘Dear Memories’ [33] aims to improve the life review process and to train the autobiographical memory, with a focus to retrieve positive specific events from the past. This protocol is based on the a life review protocol designed by Serrano et al. [25] for older adults with depressive symptomatology. LRT-MST consists of four weekly sessions on a particular lifetime period: childhood, adolescence, adulthood, and whole life span. For each period, 14 questions are designed to prompt specific positive memories. Participants are explicitly encouraged to retrieve positive specific memories to the positively stated questions. Each interview, conducted in Dutch, took approximately 1 hour and was led by a psychologist who was trained in the LRT-MST-protocol “Dear Memories”. The intervention took place at the respondent’s residence or at the hospital. The interviews were recorded on mp3 and copies were offered as a remembrance for the patients and/or their informal caregivers.

### Care as usual

During visits to the hospital, physicians and nurses provided emotional support and advice how to cope with deterioration of quality of life on an ad hoc basis. If urgent problems emerged, the patient was referred to other services, like a social worker, a psychologist or their general practitioner. In the present study, after the follow-up assessment, the patients in the waiting-list control group were offered LRT-MST as well.

### Outcome measures

Outcome measurements were collected at baseline (T0), after the intervention or after four weeks (post-treatment; T1), and after one-month post-treatment (Follow-up; T2).

The primary outcome measures were ego-integrity and despair (NEIS). Secondary outcome measures were psychological distress (HADS), quality of life (EORTC QLQ-PAL15), and the specificity of Autobiographical Memory (AMT).

The Dutch version of the Northwestern Ego-integrity Scale (NEIS) [34–37] was used. This is an 15-item questionnaire reflecting Erikson’s conception of the eight developmental phase in a person’s life [13]. It assesses despair (4 items) and ego-integrity (5 items), with higher mean subscale scores indicating more despair and ego-integrity. Participants are asked to indicate their agreement to statements such as, ‘I have reached a point where I can accept the events in my life as having been necessary’ or ‘I wish I had more time to take a different path in life’ on a scale from 1 (strongly disagree) to 6 (strongly agree). Cronbach’s alpha was .71 for Ego-integrity and .57 for Despair in the current study.

The European Organisation for Research and Treatment of Cancer Quality-of-Life Questionnaire PAL 15 (EORTC QLQ-C15-PAL) is a shortened version of the EORTC QLQ-C30 targeting cancer patients in palliative care and available in Dutch [38]. In this study we used the single item global quality of life scale (HRQOL) (How would you rate your overall quality of life during the past week?), which is scored on a 7-point scale ranging from 1, ‘very poor’, to 7, ‘excellent’. According to the EORTC guidelines, this scale was transformed to a 100-points scale with a higher score indicating a better quality of life.

A validated Dutch version of the Hospital Anxiety and Depression Scale (HADS) was used to assess psychological distress and consists of 14 items with 2 subscales: depression (HADS-D) and anxiety (HADS-A), and a total score (HADS-T). Scores on each item range from 0–3, with a total score ranging from 0–42 [39]. Cronbach’s alpha was high for both subscales and the total score, .79 (HADS-D), .83 (HADS-A) and .87 (HADS-T), in this study.The Autobiographical Memory Test (AMT) [40] was used to test the ability to retrieve a specific memory, measured in response to cue words. The AMT consists of 10 Dutch cue words, like “restaurant”, “drawing” or “success”, presented orally, one at the time. Participants are asked to retrieve a specific autobiographical memory in response to the cue word. Specific events are defined as events that occurred at a particular place and time and lasted less than one day. Answers are coded as specific, repeated (occurring more than 1 day) or extended (lasting longer than 1 day). Codes given to the memories were rated by the interviewers and checked by the coordinating researcher. Because the total number of stimulus words is 10 and each memory is rated as specific (2 points), general (1 point) or no memory (0 points), the maximum score is 20. Cronbach’s alpha, in this study, was .72.

### Sample size calculation

Based on an two-sided effect size (Cohen’s d) of .60 at one month post treatment (T2), an alpha of .05 and a statistical power (1-beta) of .80, we needed 45 patients in each study arm. With an expected drop-out rate of 20% we aimed to include 108 patients at baseline.

### Randomization

This study was a parallel-group RCT, with block randomization of 20, to make sure that the psychologists could process a gradually inflow of patients. Randomization was conducted centrally by an independent researcher (not involved in the trial) using a computer-generated randomization procedure. Patients and psychologists were aware of treatment allocation.

### Statistical analysis

Independent samples t-test and chi-square test were used to gauge whether randomization resulted in a balanced distribution of patient characteristics and outcome measures at baseline across the groups. Intention-to-treat analyses were performed. To test differences in changes from baseline to follow-up between experimental conditions, linear mixed models were used with fixed effects for group, assessment (i.e. time), and their two-way interaction, and a random intercept for subjects. If changes from baseline to follow-up between groups were significant, an independent samples t-test was performed to post-hoc assess differences between the experimental conditions immediately after the intervention or control period (T1) and follow-up assessment (T2). In that case, also effect sizes (ES) were calculated by dividing the difference between the means of the intervention and the waiting-list control group by the SD of the control group. Low, moderate and high ES were defined as, ES = .10-.30, ES = .30-.50 and ES>0.50, respectively [41]. For all statistical analyses, a p-value < .05 was considered statistically significant. Analyses were performed with SPSS 20.0 (IBM Corp., Armonk, NY USA).

## Results

### Participants

**Fig 1:**
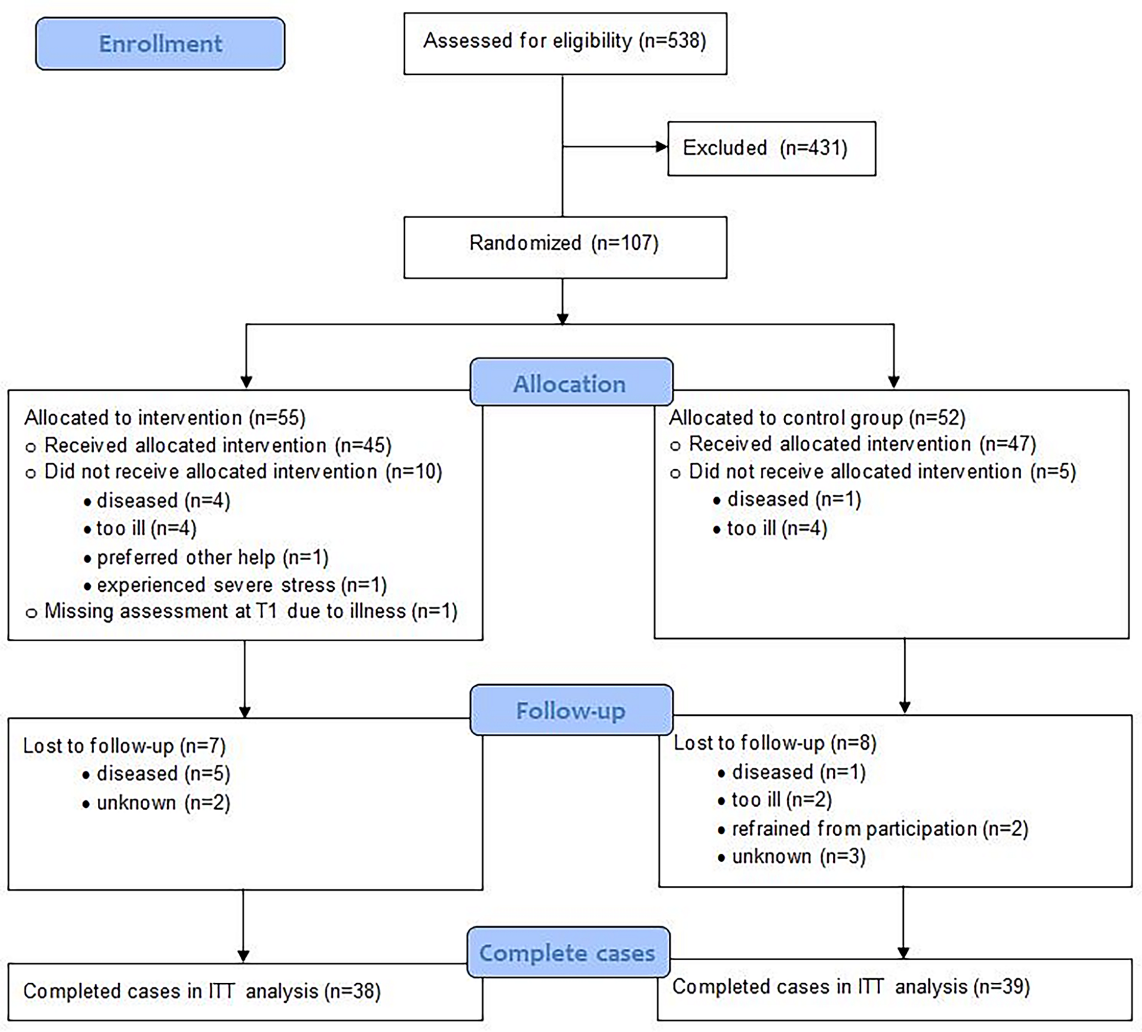
Consort flow diagram of study population. ITT, intention to treat.

During the inclusion period of 42 months (June 2010 until December 2013) a total of 538 patients were recruited. Of these patients 107 agreed to participate (20%): 55 were randomized in the intervention group, and 52 in the waiting-list control group. In total, 30 patients (28%) did not complete the study, 17 in the intervention group and 13 in the control group. Of the 55 patients in the intervention group, 39 completed the intervention (38 at home and 1 at the hospital; compliance rate 69%). Reasons for not completing the intervention were mainly disease progression and death; furthermore one patient preferred other help, one patient suffered from severe stress and didn’t want to continue, and one patient refrained from participation without an explanation. Fig 1 shows the consort flow diagram.

An overview of study population is provided in Table 1. At baseline there were no significant differences between the groups with respect to sociodemographic or clinical characteristics, or baseline outcome measures, except for the EORTC QLQ-C15-PAL Quality of Life Scale (*p* = .034). Also, the groups did not differ significantly regarding the presence of a major depressive disorder (9.3% in the intervention group versus 11.5% in the waiting-list control group; *p* = .70).

**Table 1:** Overview of patient characteristics.

	Total group (*n* = 107)		LRT (*n* = 55)		CAU (*n* = 52)		P*
Scale	Distribution	%	Distribution	%	Distribution	%	
**Gender**							0.91
*Male*	57	53.3	29	52.7	28	53.8	
*Female*	50	46.7	26	47.3	24	46.2	
**Age**							0.10
*Mean (SD)*	62.7 (9.3)		64.2 (8.5)		61.2 (9.9)		
*Range*	31–86		46–83		31–86		
**Marital status**							0.30
*Married/living together*	75	70.1	41	74.5	34	65.4	
*Divorced*	32	29.9	14	25.5	18	34.6	
**Children**							0.13
*Yes*	91	85.0	44	80.0	47	90.4	
*No*	16	15.0	11	20.0	5	9.6	
**Level of education**							0.95
*Academic education*	15	14.0	9	16.4	6	11.5	
*Higher general or vocational education*	27	25.2	14	25.5	13	25.0	
*Secondary general or vocational education*	35	32.7	18	32.7	17	32.7	
*Primary education*	28	26.2	13	23.6	15	28.8	
*None*	2	1.9	1	1.8	1	1.9	
**Religion**							1.00
*Yes*	35	32.7	18	32.7	17	32.7	
*No*	72	67.3	37	67.3	35	67.3	
**Tumor type**							0.61
*Lung cancer*	66	61.7	31	56.4	35	67.3	
*Head and neck cancer*	2	1.9	1	1.8	1	1.9	
*Hematological cancer*	23	21.5	12	21.8	11	21.2	
*Breast cancer*	5	4.7	3	5.5	2	3.8	
*Other*	11	10.3	8	14.5	3	5.8	

Note. LRT = Intervention group, CAU = Waiting-list control group—Care-as-usual, SD = Standard deviation

*chi-square test (age = independent samples t-test)

### Effectiveness of the intervention

**Fig 2:**
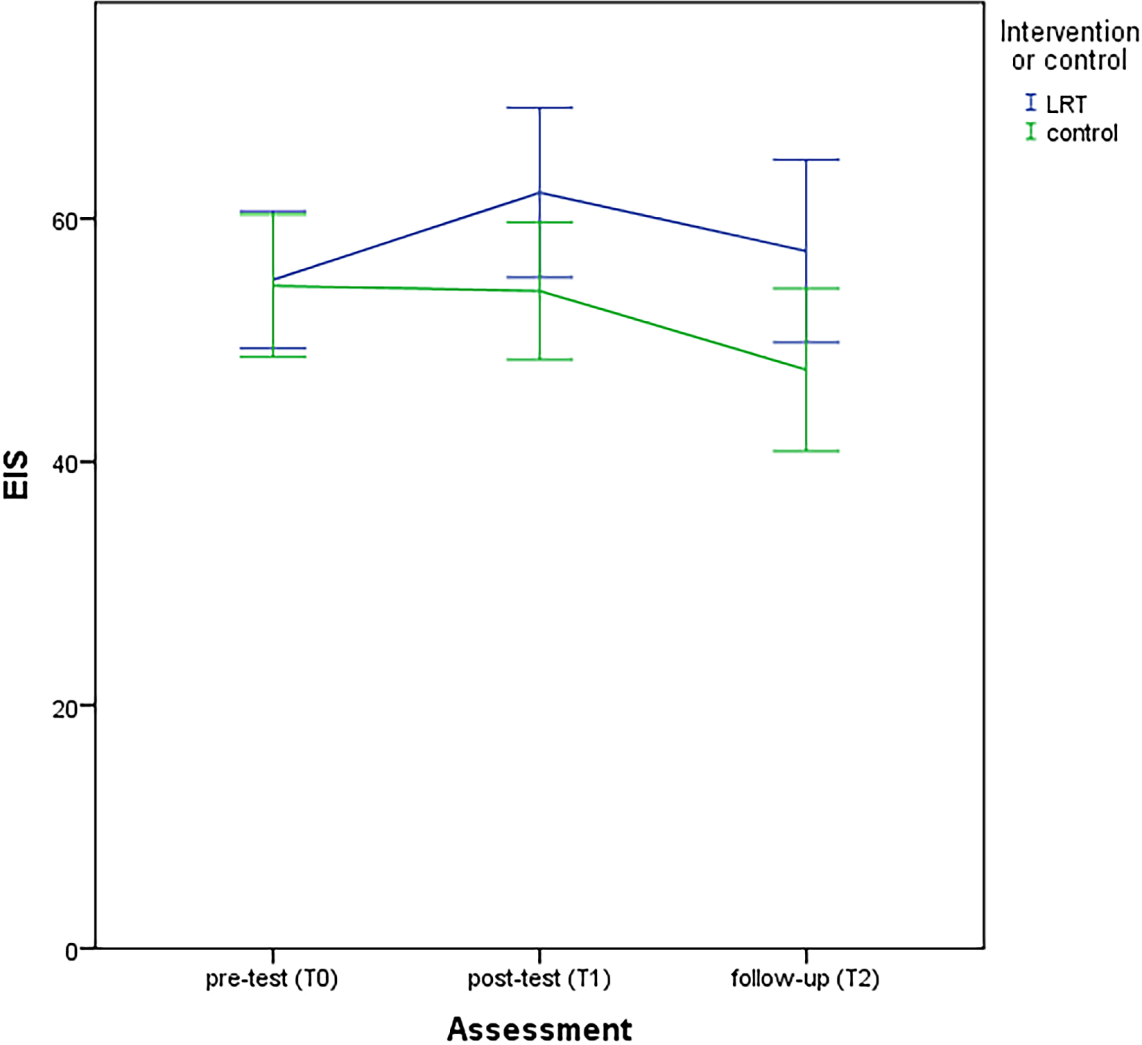
Course of NEIS: Ego-integrity scale.

Descriptive statistics of the outcome measures are provided in Table 2. The course of the ego-integrity subscale of the NEIS (Fig 2) improved significantly over time (*p* = .007) in the intervention group compared to the waiting-list control group. Ego-integrity of patients in the intervention group improved after the intervention and scores remained better at follow-up compared to baseline, while ego-integrity of patients in the waiting-list control group decreased at post-test and follow-up. No significant differences between the two groups were found regarding the course of despair (NEIS despair subscale: *p* = .89), distress (HADS-T: *p* = .30), anxiety (HADS-A: *p* = .44) depression (HADS-D: *p* = .54), quality of life (EORTC QLQ-PAL15, *p* = .058) or autobiographical memory (AMT, *p =* .070). For ego-integrity, there were moderate, borderline significant, effect sizes post-intervention (mean difference = 8.1, 95% CI: -0.71–16.9, ES = .42, t = 1.8, *df* = 88, *p* = .071) and at follow-up (mean difference = 9.8, 95% CI: -0.13–19.6, ES = .48, t = 2.0, *df* = 74, *p* = .053). In the control group, CAU comprised psychological care in 15% of the patients at T1 and 13% at T2. After the one-month follow-up assessment (T2), nine patients in the control group agreed to start the intervention (17.3%). Reasons for not starting LR-MST were among others that patients were too ill or already died.

**Table 2:** Outcomes of ego-integrity, despair, psychological distress, quality of life and AMT (over time).

	Assessment	Pre-test			Post-test					Follow-up					Interaction LLM	
Scale		*n*	M	SD	*n*	M	SD	ES	*p*ª	*n*	M	SD	*ES*	*p*ª	*F*	*p*
NEIS																
Ego-integrity	LRT	55	55.0	20.9	43	62.1	22.7	.42	.071	38	57.3	22.9	.48	.053	5.1	.007*
	CAU	52	54.5	21.0	47	54.1	19.3			38	47.6	20.3				
Despair	LRT	55	42.4	22.9	43	37.9	22.5			38	38.1	23.8			.12	.89
	CAU	51	42.4	21.2	47	39.3	20.8			36	41.8	21.6				
HADS																
Anxiety	LRT	55	5.7	4.4	44	4.6	3.8			38	5.2	4.7			.83	.44
	CAU	52	5.4	3.7	47	5.3	3.9			38	5.5	4.3				
Depression	LRT	55	6.1	4.1	44	4.9	3.7			38	5.8	4.8			.62	.54
	CAU	52	5.9	4.1	47	5.9	4.2			39	6.4	4.8				
Distress (Total)	LRT	55	11.8	7.7	44	9.5	6.9			38	11.0	8.8			1.2	.30
	CAU	52	11.3	6.9	47	11.1	6.9			38	11.6	7.7				
EORTC-PAL15																
Global Qol	LRT	55	59.4	22.9	44	68.6	18.8			37	64.9	21.4			2.9	.058
	CAU	52	68.6	21.3	47	67.0	19.2			37	65.3	21.7				
AMT	LRT	55	15.0	3.7	44	15.4	3.0			-	-	-			3.4	.070
	CAU	52	14.2	3.7	47	12.9	3.9			-	-	-				

Note. CAU = Waiting-list control group—Care-as-usual; LLM = Lineair mixed models; M = Mean; SD = Standard deviation

*F* = assessment x group; *p =* p-value

* = *p* < .05

ª = Independent samples t-test; ITT analyses were performed

## Discussion

The present study showed that LRT-MST has a positive effect on the course of ego-integrity among cancer patients in palliative care. LRT-MST had no effect on despair, psychological distress, or quality of life. There may be an effect on the specificity of the autobiographical memory.

This study confirms findings of earlier studies on the efficacy on life review interventions in patients with incurable cancer. A randomized controlled trial on the effect of life review therapy (four weekly sessions) among advanced cancer patients in a hospice in China showed positive effects on quality of life (single item) and on five of the eight scales of the Quality of life Concerns in the End of Life Questionnaire. These five scales addressed support, negative emotions, sense of alienation, existential distress, and value of life. The three non-significant scales addressed physical discomfort, food-related concerns, and healthcare concerns [22]. A trial on the effect of life review (three weekly sessions) among terminally ill cancer patients in hospitals in Japan showed a positive effect on spiritual well-being (FACIT-sp12), on psychological distress (HADS), and on aspects as hope and life completion but not on aspects as pain and symptoms (GDI) [21]. The results of these three trials indicate that life review interventions improve ego-integrity, value of life, and spiritual well-being. Whether life review interventions also improve quality of life and psychological distress remains inconclusive. Main problems comparing these three studies (although all three targeting advanced cancer patients) are the different outcome measures, but also the number of sessions of life review therapy (three or four weekly sessions) and the different residences (private home, hospice, hospital), which may have a moderating effect [17], and also the different cultures (Netherlands, China, Japan).

Future studies may provide insight into possible moderators of the intervention effect on ego-integrity such as gender, age, educational level, and clinical factors as time to death, or burden of treatment related symptoms before the intervention, as well as level of ego-integrity, quality of life or psychological distress [17].

In the present study life review therapy was combined with an autobiographical memory training. LRT-MST had a borderline effect on the autobiographical memory, which suggests that the effect of LRT-AMT on ego-integrity might be mediated by improvement of the specific autobiographical memory. Future (qualitative) studies are needed to obtain better insight into the effect of LRT-AMT on ego-integrity and possible mediators.

There are some limitations of this study that must be acknowledged. The follow-up assessment was only 1 month after treatment, and therefore we cannot generalize the results regarding a long-term effect. Also, there was a higher dropout percentage (28%) than expected (20%). It appeared to be very difficult to recruit cancer patients in palliative care timely. This is a problem also experienced in other studies, especially with advanced cancer patients [42,43]. In clinical practice it is often unclear when it is the right time to transfer a patient from treatment with intent to cure to palliative care due to for example uncertainty of patient’s disease progression, lack of accepting the failure of treatment by healthcare professionals, patients and/or patients’ family, or poor understanding of palliative care [44]. For patients, this timely offering of palliative care is important, because research showed that near end-of-life issues with meaning and purpose of life are more important than physical symptoms or physical well-being [45]. Therefore, further research and discussion in clinical practice about this topic is necessary.

Despite these limitations, it can be concluded that the LRT-AMT seems effective to improve ego-integrity among cancer patients in palliative care. Because there are currently no interventions addressing this specific and important theme, this intervention appears to be a valuable addition to psycho-oncological health care. Further research is recommended to investigate possible moderators and mediators of the intervention, the efficacy of this intervention in specific groups (for example hospice patients) and to obtain insight in the uptake and reach of the intervention in routine practice.

## Supporting information

S1 File CONSORT checklist. (DOC)

S2 File Study protocol. (DOC)
