# Supplementary material for: The efficacy of Life Review Therapy combined with Memory Specificity Training (LRT-MST) targeting cancer patients in palliative care: A randomized controlled trial
Source: PLoS One. 2018 May 15;13(5):e0197277. doi: 10.1371/journal.pone.0197277 (PMC5953483; doi:10.1371/journal.pone.0197277)
Supplement: S2 File — (DOC) [file pone.0197277.s002.doc]

**PROTOCOL TITLE:**

**Structured Life Review Using Autobiographical Retrieval Practice in Palliative Cancer Patients**

**Protocol Title**

**Structured Life Review Using Autobiographical Retrieval Practice in Depressed Palliative Cancer Patients**

| **Protocol ID** | **11510003** |
| --- | --- |
| **Short title** | **Effects of Life Review Therapy in palliative patients with cancer** |
| **Version** | **1** |
| **Date** | **17-06-2010** |
| **Coordinating investigator/project leader** | ***Prof. Dr. I.M. Verdonck – de Leeuw***  ***Dept. Of Otolaryngology – Head and Neck surgery***  ***FPP Klinische Psychologie***  [*Im.verdonck@VUmc.nl*](mailto:Im.verdonck@VUmc.nl)***; 020-444 0931*** |
| **In samenwerking met:** | ***Dr. A. Becker, lung physician***  ***Dept. Of pulmonary diseases, VU University medical center***  ***a.becker@vumc.nl*** |
|  |  |
| **Sponsor (in Dutch: verrichter/opdrachtgever)** | ***Zon Mw*** |

**PROTOCOL SIGNATURE SHEET**

| **Name** | **Signature** | **Date** |
| --- | --- | --- |
| **Legal representatives:**  **Prof. dr. P. Cuijpers,**  **Head of the department of Clinical Psychology, VU University Amsterdam**  ***Prof. dr. CR Leemans,***  **Head and Neck Surgeon, Head of the Department of Otolaryngology / Head & Neck Surgery, VU University Medical Centre**  ***Prof. dr. E.F. Smit***  **Head of Department Pulmonary Diseases, VU University Medical Centre** |  |  |
| **Principal Investigator:**  ***G. Kleijn***  ***VU University Amsterdam***  ***Department of Clinical Psychology***  ***Van der Boechorststraat 1***  ***1081 BT Amsterdam***  ***Phone: 020 598 8782*** |  |  |
| **Independent physician:**  ***Prof. dr. P.C. Huijgens*** | | |

**TABLE OF CONTENTS**

1. INTRODUCTION AND RATIONALE 7

2. OBJECTIVES 10

3. STUDY DESIGN 10

4. STUDY POPULATION 12

4.1 Population (base) 12

4.2 Inclusion criteria 12

4.3 Exclusion criteria 12

4.4 Sample size calculation 12

5. TREATMENT OF SUBJECTS 13

5.1 Investigational product/treatment 13

6. METHODS 15

6.1 Study parameters/endpoints 15

6.2 Main study parameter/endpoint 16

6.3 Withdrawal of individual subjects 17

7. SAFETY REPORTING 17

7.1 Section 10 WMO event 17

8. STATISTICAL ANALYSIS 17

9. ETHICAL CONSIDERATIONS 19

9.1 Recruitment and consent 19

9.2 Compensation for injury 19

10. ADMINISTRATIVE ASPECTS AND PUBLICATION 20

10.1 Handling and storage of data and documents 20

10.2 Amendments 20

10.3 Annual progress report 20

10.4 End of study report 20

10.5 Public disclosure and publication policy 21

12. REFERENCES 22

Appendix A: Protocol dierbare herinneringen Appendix K: Informatiebrief patiënten

Appendix B: Demografische vragenlijst Appendix L:Toestemmingsformulier

Appendix C: EORTC-QLQ-PAL15 Appendix M: Informatiebrief partners

Appendix D: AMT Appendix N: Toestemmingsbrief benadering

Appendix E: NEIS Appendix O: Verzekeringscertificaat

Appendix F: HADS

Appendix G: M.I.N.I.

Appendix H: TIC-p

Appendix:I: CRA

Appendix: J: PTGI

**SUMMARY**

**Rationale:**

Palliative cancer patients often experience feelings of reduced quality of life, sadness, hopelessness and spiritual distress, such as an evaluation of one’s past and problems with finding a new meaning in life while suffering from an incurable disease. There is an urgent need to evidence-based psychosocial intervention methods enhancing the quality of life and dying and relieving the emotional distress in palliative cancer patients in the daily practice of psychosocial care. Structured life review therapy seems to be an intervention appropriate to this need.

The autobiographical retrieval practice ‘’Dear Memories’’ focuses on bringing up positive specific events. The protocol has shown to be effective in depressed older adults, and pilot studies suggested improved quality of life in depressed and non-depressed cancer-patients and in the general population. The intervention might help palliative cancer patients in mentally understanding their prognosis and their end of life. Retrieving positive memories seem to enhance feelings of mastery, enables re-thinking and re-evaluation of life events, and may contribute to reconstruct life including the diagnosis of cancer.

According to theories on the relationship between depression and autobiographical memory depressed people have difficulties being specific in retrieving autobiographical memories.

This protocol trains patients in retrieving specific positive autobiographical memories in order to improve the quality of life.

**Relevance**

In 2008 99603 Dutch people suffered from cancer, in that same year 40993 Dutch people died from this disease. A majority of cancer patients report a reduced quality of life, and having questions about life and meaning.

**Objective**: The goal of this study is to investigate the effectiveness of a structured life review protocol named ‘’Dear Memories’’ on enhancing quality of life in palliative cancer patients and their partners, via improving the autobiographical memory. We would like to investigate possible determinants of efficacy of the intervention, such as age, gender and cultural background. If the protocol appears to be an effective intervention, it can be introduced in the regular psycho-oncological care. Spouses of these included patients will be asked to participate to study a possible effect on their quality of life: although the intervention is targeting the patients, insight from earlier studies indicate that there may be a beneficial effect on spouses as well.

**Study design:** A multicenter prospective randomised control trial with two parallel groups. Patients are assigned to either an intervention condition; receiving the life review protocol immediately after premeasurement or to a waiting list condition, receiving care-as-usual and a follow-up measurement after one month to asses long-term effectiveness.

**Study population:** A sample of 150 cancer patients without curative treatment options with a prognosis of > 3 months will be included. Exclusion criteria are cognitive impairment, inability to communicate verbally, insufficient mastery of the Dutch language and psychotic symptoms or behaviour (delusions or hallucinations).

**Intervention**: The intervention will be individually administered and consists of four weekly sessions, with every session focusing on a particular period in life – childhood, adolescence, adulthood and whole life span. For each period, 14 questions are prepared that are designed to prompt specific positive memories. For example: ‘’What is the most pleasant situation that you remember from your childhood?” The interviewer tries to get a specific view of the situation by asking for more information. Patients receive feedback on how well they are doing in retrieving specific memories.

**Main study parameters/endpoints:** Primary outcomes are the specificity of Autobiographical Memory (AMT), quality of life (EORTC QLQ-PAL15), and ego-integrity (NEIS). Secondary outcomes regard depressive symptoms (HADS; MINI). Spouses are measured on level of depressive symptoms (HADS), personal growth (PTGI), and care giving burden (CRA).

**Nature and extent of the burden and risks associated with participation, benefit and group relatedness:** Based on our experiences with depressed elderly in primary care settings, we consider the risks associated with participation to be negligible.

**1. INTRODUCTION AND RATIONALE**

In 2008 the incidence of cancer in the Netherlands was 606 per 100.000 persons. In 2008, 40993 patients died from this disease. The diagnosis cancer can have a disastrous effect on the quality of life. Treatment of cancer is frequently associated with loss of health, pain, grief, fear, and death. Especially, when there is no hope for recovery. The patient will have to adapt to live with cancer and an impending life. The quality of life is reduced in many cancer-patients, and many report having questions about life and meaning^44-45^. This shift further increases the risk of a mood ‘’adjustment’’ disorder, such as depression. Co-morbid anxiety or depression is present in about 25% of cancer patients.^1^ In a recent study including a sample of patients with palliative cancer, high levels of anxiety (34%) and particularly of depressed mood (56%) were found^3^. Besides the physical and often psychological suffering of cancer patients, patients’ primary caregivers too are more vulnerable to develop adjustment disorders. In a review about the psychological impact of cancer on patients’ partners and caregivers was found that 20-30 % of the carers develop a depressive, anxiety or adjustment disorder^4^. Their psychological problems and morbidity increases in frequency as the illness progresses, particularly when patients are suffering from advanced or terminal disease. However, carers are less likely than patients to disclose their concerns and worries and only half of those with serious psychological problems will seek help.

Psychosocial care, with its goals of relieving emotional distress and promoting well-being, is central to efforts to improve the quality of cancer patients’ lives. Psychosocial care is increasingly recognized as an essential component of the comprehensive care of patients diagnosed with cancer^5-6^. This new view on care is translated into the Nationaal Programma Kankerbestrijding (NPK). One of the NPK goals is to implement an efficient instrument before 2010 to screen for patients who are in need of psychosocial care (estimated 30-50%). Therefore, there is an urgent need to develop evidence-based psychosocial intervention methods which do not only focus on diminishing symptoms of depression but also of enhancing the quality of life and relieving the emotional distress in cancer patients.

Evidence-based psychosocial interventions for cancer patients include psycho-education, supportive therapy, relaxation training, problem-solving therapy, cognitive behavioural

therapy, couples and social skills training. In addition to depression and anxiety complaints, incurable ill cancer patients often experience sudden spiritual distress; such as that they are in search for or try to adapt their meaning or purpose in life to this new situation. They may experience an urge to make up the balance of life, recalling and evaluating their past, resolving problems from the past and assess former coping strategies. They may experience

a growing need to restore harmony with friends and relatives, or on the other side they may experience problems in relationships with family or loved ones, which may lead to a difficult farewell or unresolved relational problems.

A recent study by Ando et al.^5^ as one of the first, addressed these problems by means of a life review intervention in terminally ill cancer patients. According to Butler^7^ the life review process is a mean of reintegration and can give new significance and meaning to an individual’s life. It is defined as ‘’the progressive return to consciousness of prior experience, which can be re-evaluated with the intention of resolving and integrating past conflicts, thereby giving new significance to one’s life’. Ando et al. found significant effects of their Short-Term Life Review in depressive feelings of palliative cancer patients. Patients reviewed their lives; they re-evaluated, re-constructed and appreciated their life. Life review therapy could be a psychosocial treatment which not only addresses the emotional distress but also the spiritual distress in palliative cancer patients. The autobiographical retrieval practice ‘’Dear Memories’’ focuses on bringing up positive specific events. In previous studies with depressed older adults (not being cancer patients) the protocol proved to be effective in decreasing depressive symptoms, feelings of hopelessness and improved life satisfaction^8^.The first findings within in a Dutch population of elderly people in caring homes^9^ are positive as well.

In this study we now want to investigate the effectiveness of a structured life review protocol named ‘’Dear Memories’’ on quality of life in palliative cancer patients and their partners. Main research aim of this project is to investigate whether autobiographical retrieval practice leads to a significant enhancement of quality of life and wellbeing in palliative cancer patients with depressive symptoms.

A pilot study with the life review protocol was performed among HNC patients to investigate whether (minor) adaptations of the protocol are needed to improve the protocol to be used in (head and neck) cancer patients in the palliative phase. Temporary results suggest that the life review therapy is a relevant intervention, which helps palliative patients to reconstruct their lives and to retrieve ‘forgotten’ positive memories. Pilot respondents evaluate the intervention as pleasant and helpful.

Life review can fulfil various functions for the individual; it implies an evaluation of one’s past in order to accept negative events, resolve past conflicts, identity continuity of past and present, and eventually to find meaning in life. Butler^7^ was the first to introduce life review as a therapy. He considered life review the expression of a universal mental process reflecting the need to come to term’s with one’s life when the end is approaching. Palliative cancer patients might experience the same feelings and need to review their past when the end of their life is approaching suddenly or unexpected. Meta-analyses have proven the efficacy of life review therapy on both depression and psychological well-being in later life^14^. Studies suggest that physicians and palliative cancer patients are ambivalent about talking about death and often avoid the conversations^15-16^. This life review therapy gives a protocol in which patients are trained to retrieve specific positive memories. It has a positive effect on depressive symptoms^8-9^ and might help palliative cancer patients in mentally understanding their prognosis and their end of life. By acknowledging that death is near, patients, caregivers, and physicians can focus on clarifying patients’ priorities and relieving pain and improving symptom management^16^. Life review has some advantages above regular psychosocial treatment or support from family and loved ones. Life review gives the patient the opportunity to express emotion or distress without being concerned about the feelings of family or others.

Patients can confirm their acquisitions or roles in life, narrating their lives as they have lived them and can re-evaluate memories with emotional support from the therapist.

The protocol ‘’Dear Memories’’ is a training in retrieving specific positive memories. These memories seem to enhance feelings of mastery, of being in control of one’s own life. Feelings which are severely affected by suffering from incurable cancer. Receiving life review enables re-thinking and modifying the original purposes or goals into attainable ones, increasing the feeling of positive mood. Finally, life review may contribute for a patient to reconstruct its life being congruent with the diagnosis of cancer^8^. Our life review protocol is based on research about autobiographical memory, part of the memory where personal memories are stored. Studies on autobiographical memory retrieval show that if depressed people are presented with a cue word, they retrieve less specific and more general memories than a control group or than nondepressed persons^17-19^.This so called overgeneral memory is one of the most reliable features of memory in depression^18^. Although overgeneral memory was originally described as a stable characteristic it may in fact be open to change^20^. Serrano et al. ^8^ using ‘”Dear Memories’’ in a Spanish population of older adults found a significant increase in number of specific memories reported by the patients at post test. We want to investigate the effect of life review therapy using autobiographical memory retrieval on the specificity of retrieved memories in palliative cancer patients.

This study is the first randomised control trial using life review therapy in a palliative cancer patient sample. The first (positive) results of the Dutch translation of the protocol were within in a population of elderly people living in primary care settings^9^. In case the protocol appears to be beneficial for head and neck cancer and lung cancer patients in the palliative phase, the protocol can be introduced in psychosocial palliative care in other cancer patients as well. The proposed project relates closely to one of the goals of the National Program Cancer Control (2005-2010) to improve psychosocial care in cancer patients.

# 2. OBJECTIVES

The objective of this study is to investigate the effectiveness of the protocol ‘’Dear Memories’’ in improving quality of life within a palliative cancer patient’s population.

Furthermore the autobiographical memory is investigated whether it is malleable; we hypothesize to find that the palliative cancer patient population becomes more specific in retrieving autobiographical memory after finishing the intervention. This study will allso provide insight in possible determinants of emotional distress and efficacy of the intervention in cancer patients, such as age, gender, comorbidity, cancer treatment modality, spirituality and social support. It may be possible to develop a profile of palliative cancer patients who are

at high risk of developing depressive symptoms and to investigate which characteristics are related to efficacy of the intervention. Finally we want to investigate the psychological impact of the disease on patients’ partners as primary caregivers, the relationship between depressive symptoms of the patient and of the partner and the influence of the protocol on mental health on both patient and partner.

**3. STUDY DESIGN**

A multicenter prospective randomised controlled trial (RTC) will be carried out in two parallel groups. All patients will be individually randomly assigned to either the intervention condition, receiving the life review intervention “Dear Memories” or the waiting list condition, receiving care as usual. Questionnaires will be administered before the start of the course (t0) and 5 weeks later (t1), after the intervention has finished. A follow-up interview and assessment will be conducted one month later (t2).

Planned duration of the study is 36 months: preparation 6 months, patient inclusion and intervention: 30 months, clinical follow-up, data-management, final analysis and knowledge transfer and preparation of reports: 6 months.

In this RCT, there are two different types of patient recruitment. In the first one, the patients will receive a study information flyer from their physician. If they are interested, they will receive a phone call from the investigator providing more information about the study.  In the second type of recruitment patients are pre-selected by the investigator via weekly lists of patients visiting the departments. The investigator will contact the physician to obtain approval to approach the patient. The patient will thereafter be sent a letter signed by the physician, information about the study, and a form on which they can provide permission for the investigator to contact them. What type of patient recruitment is used depends on the logistics of the participating departments. Patients who are in the palliative phase of the disease, and who are willing to participate will be included in this study. Also, the spouses will be asked to participate in the study. One week later a pre-test will be administered en the participants will be asked to sign the informed consent (appendix L). Participants who are randomly assigned to the intervention condition will be visited by one of the two professional health care-psychologists from the Ingeborg Douwes Centre, a specialised centre for psycho-oncological care. These psychologists are experienced community workers, who are trained in the LRT-protocol “Dear Memories”. Post-tests will be administered by the principal investigator. The interviews will be recorded on a MP3-voice recorder. Copies of the digital recordings will be offered to the respondents, after finishing the interviews.

Also, the spouses will be asked to participate in the study. One week later a pre-test will be administered en the participants will be asked to sign the informed consent (appendix L). Participants who are randomly assigned to the intervention condition will be visited by one of the two professional health care-psychologists from the Ingeborg Douwes Centre, a specialised centre for psycho-oncological care. These psychologists are experienced community workers, who are trained in the LRT-protocol “Dear Memories”. Post-tests will be administered by the principal investigator. The interviews will be recorded on a MP3-voice recorder. Copies of the digital recordings will be offered to the respondents, after finishing the interviews.

# 4. STUDY POPULATION

## 4.1 Population (base)

150 palliative cancer patients and their spouses.

## 4.2 Inclusion criteria

- No curative treatment options for cancer.

- A prognosis of 3 months or more.

## 4.3 Exclusion criteria

## - Psychotic behaviour (delusions or hallucinations)

- Severe cognitive impairments

- Severe impairments in oral communication (clarity)

- Poor understanding of the Dutch language

## 4.4 Sample size calculation

The HADS is the primary outcome measure. Based on a medium standardized effect size (Cohen’s d) of 0.60 ^22^, alpha of 0.05 and a statistical power (1-beta) of 0.70, we need 51 participants in each study arm (102 in total). With a drop-out rate of 20% we will need 122 patients at baseline.

# 5. TREATMENT OF SUBJECTS

## Investigational product/treatment

The intervention “Dear Memories” is an autobiographical retrieval practice, focussing on bringing up positive specific events. By training to retrieve positive autobiographical memories, the quality of life can be increased. The life review intervention ‘’Dear Memories’’ consist of 4 weekly sessions, with every session focusing on a particular life period – childhood, adolescence, adulthood and the whole life span. For each period, 14 questions are prepared that are designed to prompt specific memories. Participants are explicitly encouraged, by means of feedback on how well they are doing, to retrieve positive specific memories to the positively stated questions. Sessions last no longer than 1,5 hour. Patients can stop at any moment, if they wish to do so. The interviews will take place at the respondent’s residence or in the hospital.

The life review therapy will be conducted by two trained mental health psychologist, from the Ingeborg Douwes Center for psycho-oncological care, experienced in working with patients in the palliative phase. The interviews will be recorded on mp3-voice recorder. Copies of the digital recordings will be offered to the respondents, after finishing the interviews.

Examples of questions include: ‘’What is the most pleasant situation that you remember from your childhood?’’; ‘’What did your mother or father do one day when you were a child that astonished you?’’; ‘’During adolescence, what moment do you remember as special because it was the first kiss you received or because you shared something special with whom you were in love?’’; ‘’Tell me about a day when you were an adolescent and you did something out of the ordinary’’, ‘’Tell me a time that you remember experiencing the most pride at work’’. Participants are explicitly encouraged, by means of feedback on how well they are doing, to retrieve positive specific memories to the positively stated questions.

Here is an example from our protocol of a positive specific and an ‘’overgeneral’’ answer to the same question. This question is one of the questions from the first interview on childhood. Interviewer: ‘’During your childhood, did you have a favourite toy?’’ Reaction from a patient: (positive specific answer): ‘’Oh yes! I head a beautiful brown teddy bear. I got it for my second birthday from my mom and dad. It was dark brown, real soft, had great dark eyes and I took it everywhere I could’’. An example of an ‘’overgeneral’’, non-specific or categorical answer on the same question by another patient: ‘’No, I can’t remember having a favourite toy. I did not

play, I did not have that much to play with either’’. After this reaction interviewer tries to get patient more specific, by means of asking some questions. Interviewer: ‘’Please try to think as

best as you can, try to go back to the age of lets say 5 years old. Can you remember what kind of games you did? Please take your time’’. If the patient than answers again he/she can’t recollect a memory, the next topic is addressed. It is possible that patients respond by expressing an ‘’overgeneral’’ memory; ‘’I can remember I played soccer with the children in the neighbourhood. Is this an answer to your question?’’

The interviewer tries to get a clearer view of the situation by asking for more detailed information.

The interviewer is not allowed to be very directive and should not pose more than one or two questions to get a more specific memory. They receive a structured standardized training in using the protocol before carrying out the life review therapy among palliative patients. They will be coached and supervised by the applicants of this study, experienced researchers on this topic. **(Appendix A)**

# 6. METHODS

## Study parameters/endpoints

## Patients are measured on the following parameters: Autobiographical Memory Test (AMT), European Organisation for Research and Treatment of Cancer Quality of Life Questionnaire Cancer for 1) Patients in palliative care (EORTC QLQ-PAL 15), and the North western Ego Integrity Scale (NEIS); secondary measures are Hospital Anxiety and Depression Scale (HADS) and Mini-International Neuropsychiatric Interview (M.I.N.I.). The patients’ partners are measured on the following parameters: Hospital Anxiety and Depression Scale (HADS), Post Traumatic Growth Inventory (PTGI-VUmc) and the Caregiver Reaction Assessment Scale (CRA). A general demographic questionnaire is administered on both participants and their partners.

## 6.2 Main study parameter/endpoint

The general demographic questionnaire **(Appendix B)** consists of 7 items, about age, heritage, religion, marital status and education.

The EORTC QLQ-PAL15 **(Appendix C)** (European Organisation for Research and Treatment of Cancer Quality of Life Questionnaire Cancer for patients in palliative care) is a cancer-specific, patient-based questionnaire^46^. The questionnaire includes a global HRQOL scale and comprises 2 functional scales: physical functioning, emotional functioning, and 3 symptom scales (fatigue, nausea/vomiting and pain) and 6 single items relating to dyspnoea, insomnia, loss of appetite, constipation, diarrhoea and financial difficulties).

The Autobiographical Memory Test (AMT) **(Appendix D)** measures respondents’ ability to retrieve a specific memory in response to a cue word^48^. Respondents are asked to retrieve a specific memory to a self-reference personality characteristic or trait,

such as creative, friendly or guilty. A specific memory is defined as a discrete episode, lasting no longer than a single day that happened to the participant. If the memory happened on a

number of occasions, it is coded as general. The cue words will be presented to the respondents orally. At pre measurement the following 10 words are presented; drawing, scared, success, bicycle, restaurant, journey, act, angry, school and animal. At post measurement the following 10 words are presented; present, television, boring, train, enthusiastic, telephone, assignment, disappointed, funny, and trip. Words are presented in a fixed, alternating order.

To ensure that participants understand the instructions, examples are provided. If for

example the cue word is proud, the respondent might say “I was really proud last Wednesday when my daughter gave birth to my first grandchild’’. That would be a specific personal memory because it referred to a particular event on a particular day when you displayed the trait. In an example as ‘’I always get proud when I see my grand children’’, the answer is not coded as a specific personal memory because it did not refer to any specific event but rather to ‘’visits of family members in general’’. The respondents are instructed to think of a specific personal memory – a time when they displayed the trait in question. Codes given to the memories were rated by the interviewers and controlled by the coordinating researcher. Because the total number of stimulus words is 10 and each memory is rated as general or specific, the maximum score for either category is 10. When for example the patient gives to 4 cue words a categorical or ‘’overgeneral’’ response and to 6 a specific memory is recalled, than patients total score on specific memories recalled is 6.

The North-western Ego Integrity Scale (NEIS) **(Appendix E)** is a questionnaire about finding meaning in life, restructuring the self and accepting life as it occurred^47^.

The level of depressive symptoms is the total score on the Hospital Anxiety and Depression

Scale (HADS) **(Appendix F)**. This is a screening questionnaire for depression and anxiety and is frequently used in cancer patients. It was specially designed for use in the medical ill and has been widely used before in patients with head and neck cancer. It consists of 14 questions, seven for anxiety and seven for depression. An example question is: ‘Lately, I get a sort of frightened feeling, like ‘butterflies’ in the stomach’. Each answer is rated on a 4-point scale, ranging from 0 (seldom or never) to 3 (always or almost always), scores range from 0 to 21 on each subscale. The generally used cut-off score for a clinically significant level of depressive symptoms is 7. The HADS is designed to be used in severe ill patients, and is a frequently used questionnaire in severe ill head and neck cancer patients^23^.

The Mini-International Neuropsychiatric Interview (M.I.N.I.) **(Appendix G)** will be used to assess the presence of Major Depressive Disorder (MDD) current and lifetime, according to DSM-IV criteria. In this study the Dutch translation of the clinician-rated (CR) version of the M.I.N.I. will be used. Validation research proved good to very good kappa values when the M.I.N.I.- CR was compared to the Structured Clinical Interview DSM-III-R-patient version (SCID-P) and the Composite International Diagnostic Interview for ICD-10 (CIDI)^24^.

The TIC-p (**Appendix H**) is a questionnaire about illness, the use of medication and medical services (specialist, family doctor), the use of services in the field of psychological care, sporting/fitness careprograms, nutritional programs and the help of significant others.

The Caregivers Reaction Assessment Scale (CRA) **(Appendix I)** is a self-rating scale that covers five domains: self-esteem, family support, finances, daily schedule, and health.

The Post Traumatic Growth Inventory (PTGI) **(Appendix J)** is a 21-item scale about positive outcomes of negative (traumatic) events, including factors of new possibilities, relating to others, personal strength, spiritual change, and appreciation of Life.

## Withdrawal of individual subjects

If during the study a patient or a spouse for whatever reason no longer wishes to participate, he or she can withdraw consent at any time without consequences for treatment in the VUmc or NKI/AvL. The researcher can decide to stop the participation on medical grounds.

# 7 SAFETY REPORTING

## Section 10 WMO event

In accordance to section 10, subsection 1, of the WMO, the investigator will inform the subjects and the reviewing accredited METC if anything occurs, on the basis of which it appears that the disadvantages of participation may be significantly greater than was foreseen in the research proposal. The study will be suspended pending further review by the accredited METC, except insofar as suspension would jeopardise the subjects’ health. The investigator will take care that all subjects are kept informed.

**8 STATISTICAL ANALYSIS**

**8.1 Descriptive statistics**

Descriptive statistics will be generated for the range of outcome variables, in particular to gauge whether randomisation resulted in a balanced distribution of patients characteristics across the experimental conditions.

## 8.2 Univariate analysis

The primary clinical outcome, intervention effect on memory (AMT) and quality of life (EORTC-QLQ-PAL15; NEIS; PTGI), will be evaluated with intention-to-treat logistic regression analysis to produce the odds ration (OR).

## 8.3 Multivariate analysis

Repeated measures ANOVA will be used to determine the efficacy of intervention for continuous outcomes such as changes in quality of life. Longitudinal changes over time in these variables will also be evaluation over all time points simultaneously using generalized estimating equations (GEEs). We will use multivariate variance analysis for repeated measurements to test whether the outcome measures increase/chance significantly more in the experimental group than in the control group. All the analyses are to be conducted on the intention-to-treat principle. If missing values emerge at t1 and t2 on the outcome measures as a result of drop-out, they will be imputed according to the last-observation-carried-forward principle or in a more advanced fashion (regression imputation or multiple imputation, both as implemented in Stata) using available baseline data from the responders as well as the non responders. This means that not every non responder received the same post-test score, but post-test score will be dependent on the particular characteristics as defined by baseline (eg gender, age).

# 9 ETHICAL CONSIDERATIONS

## Recruitment and consent

There are two different types of patient recruitment. In the first one, the patients will receive a study information flyer from their physician. If they are interested, they will receive a phone call from the investigator providing more information about the study.  In the second type of recruitment patients are pre-selected by the investigator via weekly lists of patients visiting the departments. The investigator will contact the physician to obtain approval to approach the patient. The patient will thereafter be sent a letter signed by the physician, information about the study, and a form on which they can provide permission for the investigator to contact them. What type of patient recruitment is used depends on the logistics of the participating departments. Patients who are in the palliative phase of the disease, and who are willing to participate will be included in this study. Spouses of these included patients will be asked to participate to study a possible effect on their quality of life: although the intervention is targeting the patients, insight from earlier studies reveals that there may be a beneficial effect on spouses as well.

**Feasability**

Since 2011, we started inclusion of participants for this study in several departments. Within 6 months, 11 people with depressive symptoms participated. This is only a small percentage of all eligible participants (including non-depressive) in that period. Moreover, our recent meta-analysis shows that the percentage of cancer patients who show depressive symptoms on the HADS is relatively small compared to the total group of cancer patients. On the basis of this recent data, we expect to include approximately 150 patients in the remaining 1 ½ year of our study.

## 9.2 Compensation for injury

For possible injury due to the study there is an insurance policy with the insurance company Onderlinge Waarborgmaatschappij Centramed, P.O box 191, 2270 AD Voorburg. An insurance for patients participating in the study is available in accordance with the legal requirements in the Netherlands (Article 7 WMO and the Measure regarding Compulsory Insurance for Clinical Research in Humans of 23th June 2003). This insurance provides cover for damage to research subjects through injury or death caused by the study: € 450.000,-- (i.e. four hundred and fifty thousand Euro) for death or injury for each subject who participates in the Research; € 3.500.000,-- (i.e. three million five hundred thousand Euro) for death or injury for all subjects who participate in the Research; € 5.000.000,-- (i.e. five million Euro) for the total damage incurred by the organisation for all damage disclosed by scientific research. The insurance applies to the damage that becomes apparent during the study or within 4 years after the end of the study (Appendix O).

# 10 ADMINISTRATIVE ASPECTS AND PUBLICATION

# 10.1 Handling and storage of data and documents

The investigator or a qualified employee will enter all relevant study data onto Informed Consent Forms *(ICF)* in accordance with the instructions provided. The investigator must sign the CRFs thereby stating that he/she takes responsibility for the accuracy of the data and will be responsible for the data collection and data management. Patient data and data from questionnaires of patients will be coded directly after being collected and will be entered into a statistical database to ensure accuracy and completeness of the data. The confidentially of records that could identify subjects is protected. The questionnaires will be archived and preserved for 5 years before they will be destroyed.

## 10.2 Amendments

Amendments are changes made to the research after a favourable opinion by the accredited METC has been given. All amendments will be notified to the METC that gave a favourable opinion.

## 10.3 Annual progress report

The sponsor/investigator will submit a summary of the progress of the trial to the accredited METC once a year. Information will be provided on the date of inclusion of the first subject, numbers of subjects included and numbers of subjects that have completed the trial, serious adverse events/ serious adverse reactions, other problems, and amendments.

## 10.4 End of study report

The investigator will notify the accredited METC of the end of the study within a period of 8 weeks. The end of the study is defined as the last patient’s last visit. In case the study is ended prematurely, the investigator will notify the accredited METC, including the reasons for the premature termination. Within one year after the end of the study, the investigator/sponsor will submit a final study report with the results of the study, including any publications/abstracts of the study, to the accredited METC.

## 10.5 Public disclosure and publication policy

The project will be embedded in the VUmc Cancer Centre Amsterdam / Research Institute for Cancer and Immunology (CCA/V-ICI) and the Institute for Research in Extramural Medicine (EMGO). Knowledge transfer of the results of the proposed project includes submitting papers to (inter)national peer-reviewed journals, proceedings, and news letters and presenting papers at national and international conferences, both in early pilot stages and after conclusion of the project.

# REFERENCES

1. Kleijn, G., Krebber, A.M.H., Buffart, L., Riepma, I.C., Straten van, A. and Verdonck-de Leeuw, I.M. (2012). Prevalence of depression in terminally ill cancer patients: a meta-analysis. In preparation.

3. Andersen, B.L. (1992). Psychological interventions for cancer patients to enhance the quality of life. *Journal of Consulting and Clinical Psychology, 60*, 552-568.

4. Newell, S., Sanson-Fisher, R. and Savolien, N. (2002). Systematic review of psychological therapies for cancer patients: Overview and recommendations for future research. *Journal of the National Cancer Institute,* 94, 558 – 584.

5. Pitceathly, C.& Maguire, P. (2003). The psychological impact of cancer on patients’ partners and other key relatives: a review. *European Journal of Cancer,* 39, 1517 - 1524.

6. Ando, M., Morita, T., Okamoto, T., Ninosaka, Y. (2008). One-week short-term Life review can improve spiritual well-being of terminally ill cancer patients. *Psycho-Oncology,* 17, 885 – 890

7. Butler, R.N. (1963). The life-review: an interpretation of reminiscence in the aged. *Psychiatry,* 26, 65 – 76.

8. Serrano, J.P., Latorre, J.M. and Gatz, M. (2004). Life-review therapy using autobiographical retrieval practice for older adults with depressive symptomatology. *Psychology and Aging*, 19, 272 – 277.

9. Pot, A.M., Melenhorst, A.S., Onrust, S. & Bohlmeijer, E. (2008). (Cost)effectiveness of life review for older adults: design of a randomized controlled trial. *BMC Public Health, 8:* 211.

10. Kennisnetwerk Integrale Kankercentra (IKC, 2006). [www.ikcnet.nl](http://www.ikcnet.nl/)

12. Teunissen SC, Wesker W, Kruitwagen C, de Haes HC, Voest EE, de Graeff A. Symptom prevalence in patients with incurable cancer: a systematic review. J Pain Symptom Manage. 2007;34(1):94-104.

14. Bohlmeijer, E.T., Cuijpers, P., Smit, F. (2003). Effects of reminiscence on depression and life-satisfaction in the elderly: a meta-analysis. *International Journal of Geriatric Psychiatry,* 18, 1088 – 1094.

15. Leydon, G.M., Boulton, M., Moynihan, C. et al. (2000). Cancer patient’ information needs and information seeling behavior: in depth interview study. *BMJ,* 320, 909-913.

16. Wright, A.A., Zhang, B., Ray, A. et al. (2008). Associations Between End-of-life Discussions, Patient Mental Health, Medical Care Near Death, and Caregiver Bereavement Adjustment. *JAMA*, 14, 1665-1973.

17. Butler, R.N. (1974). Succesfull aging and the role of the life review. *Journal American Geriatric Society*, 22, 529-535.

18. Kuyken, W. and Dalgleish, T. (1995). Autobiographical Memory and depression. *British Journal of Clinical Psychology*, 34, 89-92.

19. Williams, J.M.G. and Scott, J. (1988). Autobiographical memory in depression. *Psychological Medicine*, 18, 689-695.

20. Brittlebank, A.D., Scott, J., Williams, J.M.G. and Ferrier, I.N. (1993). Autobiographical memory in depression: State or trait marker? *British Journal of Psychiatry*, 162, 118-121.

21. Van ’t Veer-Tazelaar PA, van Marwijk HWJ, van Oppen P, van Hout HPJ, van der Horst HE, Cuijpers P, Smit F, Beekman ATF (2008). Stepped-care prevention of anxiety and depression in late life: a randomized controlled trial. Archives of General Psychiatry, 66, 297-304.

22. Lipsey, M.W., Wilson, D.B. (1993) The efficacy of psychological, educational, and behavioural treatment. Confirmation from meta-analysis. Am Psychol. 48(12):1181-209.

23. Katz, M.R., Kopek, N., Waldron, J., Devins, G.M. _ Tomlinson, G. (2004). Screening for depression in head and neck cancer. *Psycho-oncology,* 13, 269 – 280.

24. Sheehan, D.V., Lecrubier, Y., Sheehan, K.H., Amorim, P., Janavs, J., Weiler, E., Hergueta, T., Baker, R. & Dunbar, G.C. (1998). The MINI-Interantional Neuropsychiatric Interview (M.I.N.I.): The development and validation of a Structured Diagnostic Psychiatric Interview for DSM-IV and ICD-10. *Journal of Clinical Psychiatry,* 59, 22 – 33.

25. De Bree R, Veenvliet P, Van Driel M, Verdonck-de Leeuw IM, Goverde K, Koense YJ, Leemans CR. (2007) Snellere diagnostiek van hoofd-halskanker: Optimalisatie van zorg bij patiënten met hoofd-halskanker. *Medisch Contact*, 62: 344-347.

26. De Bree R, Verdonck-de Leeuw IM, Keizer AL, Houffelaar A, CR Leemans. Touch screen computer-assisted health-related quality of life and distress data collection in head and neck cancer patients. Clin Otolaryngol (in press).

27. Verdonck-de Leeuw IM, de Bree R, Leemans CR. Patient reported outcome (PRO) bij patiënten opgenomen voor palliatieve behandeling van hoofd-halskanker (submitted).

28. Carlson, L.E. en Butz, B.D. (2004). Efficacy and medical offset of psychosocial interventions in cancer care: The case for economic analyses. *Psycho-Oncology,* 10, 156 – 165.

29. Conway, M.A. an Bekerian, D.A. (1987). Organization in autobiographical memory. *Memory & Cognition, 15,* 119 – 132.

30. Erikson, E.H. (1963). *Childhood and Society.* New York: Norton.

Evans, D.L., Charney, D.S., Lewis, L., Golden, R.N., Gorman, J.M., Krishan, K.R., et al. (2005). Mood disorders in the medically ill: Scientific review and recommendations. *Biological Psychiatry, 58,* 175 – 189.

31. Fry, P.S. and Barker, L.A. (2002). *Female survivors of abuse and violence: the influence of storytelling reminiscence on perceptions of self-efficacy, ego strength and self-esteem*. In J.D. Webster and B.K. Haight (Eds). *Critical Advances in Reminiscence Work*, New York: Springer Publishing Company.

32. Garland, J. and Garland, C. (2001). *Life review in health and social care, a practitioner’s guide.* East Sussex: Brunner-Routledge.

33. Haight, B.K., Coleman, P. and Lord, K. (1995). The linchpins of successful life review: sstructure, evaluation and individuality. In K. Haight and J. Webster (Eds). *The art and science of reminiscing.* New York: Taylor and Francis.

34. Haight, B.K., Michel, Y., and Hendrix, S. (1998). Life review: Preventing despair in newly relocated nursing home residents: Short- and long-term effects. *International Journal of Aging and Human Development,* 47, 119 – 142.

35. Hopko, D.R. and Lejuez, C.W. (2008). *A cancer patient’s guide to overcoming depression and anxiety: getting through treatment and getting back to your life.* New York: New Harbinger Press.

36. McAdams, D.P., Reynolds, J., Lewis, M.L., Patten, A. en Bouwman, P. (2001). When bad things turn good and good things turn bad: sequences of redemption and contamination in life narrative, and their relation to psychological adaptation in midlife adults and in students. *Personality and Social Psychology Bulletin,* 27, 472 – 483.

37. Moorey, S., Greer, S., Bliss, J. en Law, M. (1998). A comparison of adjuvant psychological therapy and supportive counselling in patients with cancer. *Psycho-Oncology,* 7, 218-228.

38. Vereniging van Integrale Kankercentra (VIKC, 2006). *Landelijke Richtlijn Depressie Versie 1.1*. [www.oncoline.nl](http://www.oncoline.nl/).

39. Watt, L.M. en Cappeliez, P. (2000). Integrative and instrumental reminiscence therapies for depression in older adults: intervention strategies and treatment effectiveness. *Aging and Mental Health,* 4, 166 – 177.

40. Williams, J.M.G., Ellis, N.C., Tyers, C., MacLeod, A.K. and Rose, G. (1996). Specificity of autobiographical memory and imageability of the future. *Memory & Cognition, 24,* 116 – 125.

41. Williams, J.M.G., Barnofer, T., Crane, C., Raes, D.H., Watkins, E. & Dalgleish, T. (2007). Autobiographical memory specificity and emotional disorder. *Psychological, 133,* 122 – 148.

42. Wong, P. (1995). *The processes of adaptive reminiscence*. Washington: Taylor and Francis.

43. Birren, J.E. and Deutchman, D.E. (1991). *Guiding autobiography groups for older adults: exploring the fabric of life.* The John Hopkins University Press.

44. Henoch, I, Danielson, E. (2009). Existential concerns among patients with cancer and interventions to meet them: an integrative literature review, *Psycho-Oncology,* *18*,225-236.

45. Soothill K, Morris SM, et al. (2001). The significant unmet needs of cancer patients: probing psychosocial concerns. *Supportice care in cancer,* *9*(L), 597-605.

46. Groenvold M., Petersen M.A., Aaronson N.K., Arraras J.I., Blazeby J.I., Bottomley A., Fayers P.M., Graeff A. de., Hammerlid E., Kaasa S., Sprangers M.A.G., Bjorner J.B., (2006). The development of the EORTC QLQ-C15-PAL: a shortened questionnaire for cancer patients in palliative care. *European Journal of Cancer, 42*, 55-64.

47. Webster, J.D. (2010). Wisdom and positive psychosocial values in young adulthood. *Journal of Adult Development, 17*, 70-80.49.

48. Van Vreeswijk, F., and De Wilde, E.J. (2004). Autobiographical memory specificity, psychopathology, depressed mood and the use of the Autobiographical Memory Test: a meta-analysis. *Behavioral Research and Therapy, 42,* 731-743.

**AppendixA: Protocol “Dierbare herinneringen”**

***Dierbare herinneringen***

**Protocol voor individuele life-review therapie gebaseerd op autobiografische oefening**

**Patiënten met kanker met depressieve klachten**

Een geadapteerde versie voor het onderzoek naar de Levensverhaal methode bij kanker patiënten in de palliatieve fase.

“Structured life review using autobiographical retrieval practice in palliative cancer patients”.

Ernst Bohlmeijer, Bas Steunenberg, Roeslan Leontjevas, Margje Mahler, Riët Daniël en Debby Gerritsen

**Colofon**

*Auteurs*

E. Bohlmeijer

B. Steunenberg

R. Leontjevas

M. Mahler

R. Daniël

D. Gerritsen

*Realisatie*

Dit protocol werd ontwikkeld in het kader van een samenwerkingsproject tussen het VUmc, NKI/AVL, de Vrije Universiteit en de Universiteit Twente.

© 2010 E.T. Bohlmeijer

Dit protocol mag onder vermelding van bron vrij worden toegepast in zorg. Alle (onderzoeks)rechten voorbehouden. Niets uit deze uitgave mag worden aangepast zonder voorafgaande schriftelijke toestemming van de auteur.

**Inhoudsopgave**

[1. Het protocol](#__RefHeading___Toc229545085)

[1.1 Algemene toelichting](#__RefHeading___Toc229545086)

[1.1.1 Oriënterend gesprek](#__RefHeading___Toc229545087)

[1.1.2 Tweede en volgende gesprekken](#__RefHeading___Toc229545088)

[1.2 Thema’s en vragen](#__RefHeading___Toc229545089)

[1.2.1 De kindertijd (0-12 jaar)](#__RefHeading___Toc229545090)

[1.2.2 De jeugd (12 – 18 jaar) **Fout! Bladwijzer niet gedefinieerd.**](#__RefHeading___Toc229545100)

[1.2.3 De volwassenheid](#__RefHeading___Toc229545103)

[1.2.4 Het leven in het algemeen](#__RefHeading___Toc229545126)

[2. Aandachtspunten bij de uitvoering](#__RefHeading___Toc229545135)

# 1 Het protocol

## Algemene toelichting

Het oorspronkelijke protocol *Dierbare Herinneringen* is onderdeel van de tweede behandelmodule van het zorgprogramma *Doen bij Depressie* voor ouderen met depressieve klachten. Na minimale aanpassingen wordt het protocol momenteel getoetst als therapeutische methode voor kankerpatiënten in de palliatieve fase. Dit is de geadapteerde versie.

*Doelen van de therapie*

De doelstelling van de Dierbare Herinneringen Therapie (DHT) is dat patiënten met kanker in de palliatieve fase op een gestructureerde wijze specifieke positieve herinneringen ophalen aan hun leven. Het accent wordt vooral gelegd op het leren/ trainen om specifieke positieve herinneringen op te halen en minder op betekenisgeving of integreren van positieve en negatieve ervaringen.

Bij *Dierbare Herinneringen* is het uitgangspunt dat de herinneringen aan het eigen leven van een patiënt met depressieve symptomen, globale, negatief gekleurde verhalen zijn. Er is geen toegang meer tot allerlei specifieke, positieve gebeurtenissen in het eigen leven. Door het ophalen van deze specifieke positieve herinneringen worden uitzonderingen op de negatieve verhalen over het eigen leven versterkt en wordt een positieve zelfidentiteit versterkt. De negatieve herinneringen en negatieve interpretaties worden minder dominant. De depressieve klachten zullen daardoor afnemen. In narratieve termen: de uitzonderingen op de dominante, negatieve levensverhalen worden actief bevraagd en versterkt of verdikt.

*Opbouw van Dierbare Herinneringen*

In de life review gesprekken worden de respondenten getraind volgens het protocol in het specificeren van hun herinneringen. In beginsel worden 4 behandelgesprekken van ongeveer 60 tot 90 minuten aanbevolen. De opbouw is als volgt:

Gesprek 1: Een oriënterend gesprek, tijdens de voormeting

Gesprek 2: Training in ophalen van herinneringen uit de kindertijd (0-12 jaar)

Gesprek 3: Training in ophalen van herinneringen uit de jeugd (12 -18 jaar)

Gesprek 4: Training in ophalen van herinneringen volwassenheid

Gesprek 5: Training in ophalen van herinneringen uit het leven in het algemeen en afsluitend

gesprek

De paragrafen 1.1.1. tot en met 1.2.4. gaan dieper in op de opbouw van de afzonderlijke behandelgesprekken en geven suggesties voor te stellen vragen. Hoofdstuk 2 bevat aanbevelingen en adviezen over de toe te passen gesprekstechnieken en de benodigde vaardigheden.

***Is DHT altijd toe te passen?***

Wanneer de patiënt het pertinent niet ziet zitten, is het uiteraard van belang om dit te respecteren. Andere patiënten zullen tegensputteren of aarzelen. In dat geval kan het de moeite waard zijn om voor te stellen om het te proberen:

‘We spreken af dat we beginnen en als u na een tijdje niet verder wilt, dan stoppen we er direct mee, is dat goed?’

Het kan zijn dat patiënten er niet iets positiefs of ‘helpends’ aan ervaren of dat de gesprekken onrust veroorzaken. Het spreekt voor zich om in dat geval niet verder aan te dringen. De patiënt heeft op elk moment gedurende de therapie de vrije keuze om te stoppen, zonder opgave van reden.

### Voormeting en oriënterend gesprek (60 min)

Voor het onderzoek naar de effecten van het protocol “Dierbare Herinneringen” wordt er eerst een voormeting verricht. Dit gesprek zal tevens een oriënterend gesprek zijn waarbij een genealogie gemaakt zal worden ter voorbereiding op de overige vier gesprekken. De genealogie omvat een beknopt overzicht van de samenstelling van gezin van herkomst, opleiding, werk, samenstelling eigen gezin, woonplekken en belangrijke life events.

***De doelen van het oriënterende gesprek zijn:***

1. bespreken van doel en opzet van DHT.
2. inschatten of DHT aansluit bij de behoeften en (cognitieve) mogelijkheden van de patiënt;
3. motiveren en uitleg rationale, achterliggende theorie en werkingsmechanismen van DHT;
4. zich oriënteren op het leven van de patiënt en daarmee op levensfasen, relaties en gebeurtenissen die geschikt kunnen zijn als bron van positieve herinneringen;
5. bepalen therapiedoelen, planning en opbouw van de volgende gesprekken, in samenspraak met de patiënt.
6. (wanneer mogelijk) wat eerste vragen stellen en oefenen. Eventueel huiswerk bepalen en/of oefeningen bedenken die in het activiteitenplan kunnen worden opgenomen.

***Het voeren van het oriënterende gesprek***

*Introductie*

Begin het eerste gesprek met een korte introductie over de reden waarom je de patiënt wilt spreken. Vertel in de voor de patiënt begrijpelijke taal over de achterliggende theorie en werkingsmechanismen van DHT. Bijvoorbeeld:

‘Uit de resultaten van de vragenlijsten die u hebt ingevuld is gebleken dat u last heeft van somberheids e/of angstklachten. Het blijkt nu zo te zijn dat mensen die somber zijn, moeite hebben om zich positieve gebeurtenissen te herinneren. Ze zijn de goede herinneringen als het ware kwijt. Daarom lijkt het alsof het leven alleen maar naar is geweest. Maar het blijkt ook dat je wel weer bij die goede herinneringen kunt komen door dat te trainen. Het blijkt dat wanneer iemand getraind is in het ophalen van goede herinneringen, de stemming kan verbeteren. Er is hiervoor een speciale methode ontwikkeld die echt blijkt te werken en waarin ik ook speciaal ben getraind. Ik denk dat deze methode goed bij u toepasbaar is, zodat we samen naar verbetering van uw stemming kunnen gaan.’

Vertel dat het belangrijk is om specifieke positieve herinneringen op te halen en geen algemene herinneringen. Maak een voorbeeld van een specifieke herinnering versus een algemene herinnering. Een algemene herinnering is bijvoorbeeld dat de grote broer van een patiënt heel aardig was voor hem/haar als kind, een specifieke herinnering is dat die broer van zijn eerst verdiende geld een bal voor haar heeft gekocht.

‘Ik zou dat heel graag eens met u willen proberen. We gaan dus juist op zoek naar specifieke momenten in u leven die voor u dierbaar zijn.’

*Oriëntatie op het leven van de patiënt*

Leg uit dat je je eerst moet voorbereiden en daarom wilt oriënteren op het leven van de patiënt. Breng globaal de kindertijd (0-12), de jeugd (12-18) en de volwassenheid in kaart en vraag naar belangrijke personen. Deze inhoudelijke oriëntatie zal tevens belangrijke informatie geven voor de inschatting of *DHT* aansluit bij de patiënt.

*Oefenen en afsluiting*

Probeer verder even te oefenen door vragen te stellen uit de periode die als meest positief naar voren is gekomen tijdens de oriëntatie op het leven van de patiënt. Probeer daarmee de patiënt te motiveren en een positieve stemming te bewerkstelligen. Bespreek – wanneer mogelijk – de therapiedoelen en de planning met de patiënt. Sluit het gesprek af met een nieuwe afspraak en – wanneer dit mogelijk is – met een huiswerkopdracht. De patiënt kan zich bijvoorbeeld voorbereiden op het volgende gesprek door na te denken over de te bespreken periode en eventueel hulpmateriaal (bijvoorbeeld, foto’s) te zoeken.

***Voorbereiding van de volgende gesprekken***

Maak voor jezelf een globale planning voor de volgende gesprekken. Op de volgende pagina’s van dit protocol staat een aantal te stellen vragen afgedrukt. Per levensfase zijn enkele vragen voorgeselecteerd, dit zijn steeds de *eerste* 3 vragen, behalve bij het laatste gesprek. In dat gesprek is de eerste vraag en zijn de *laatste* 3 vragen voorgeselecteerd. Houd de volgorde ‘kindertijd’, ‘jeugd’, ‘volwassenheid’ en ‘leven in het algemeen’ aan en behandel in één gesprek niet meer dan één fase.

### Tweede en volgende gesprekken (steeds 60-90 min)

In de volgende gesprekken train je gedurende ongeveer 60 tot 90 minuten de patiënt om op een gestructureerde wijze specifieke positieve herinneringen op te halen uit diverse perioden van zijn of haar leven. Het is belangrijk om per gesprek meerdere specifieke gebeurtenissen op te halen en niet heel diep op één van de gebeurtenissen in te gaan. Hoe meer vragen, hoe beter. Houd per vraag 3 tot 4 minuten aan. Geef per vraag feedback over de specificiteit van de herinnering. Check geregeld of iemand in de goede levensfase zit. Foto’s kunnen goed helpen bij het ophalen van specifieke herinneringen. Geef daarom altijd ruimte om foto’s te bekijken.

Hoofdstuk 2 bevat aandachtspunten bij de uitvoering van DHT.

**Inhoud van de gesprekken:**

1) een korte inleiding en herhaling van de rationale,

2) – eventueel – een korte bespreking van een huiswerkopdracht,

3) de eigenlijke training in het ophalen van de herinneringen (8 á 10 vragen)

4) evaluatie en feedback.

5) bespreking van een nieuwe huiswerkopdracht (wanneer van toepassing) en planning van een volgend gesprek.

In het laatste gesprek rond je de DHT af en kun je eventueel afspraken maken voor mogelijke (zelfstandige) stappen in de toekomst.

We bevelen aan om ieder gesprek te beginnen met wat algemene vragen die betrekking hebben op de levensfase waar je vragen over gaat stellen. Zo ontstaat er context en draait het autobiografisch geheugen ‘warm’. Na vijf á tien minuten kun je dan de eerste vraag naar een specifieke positieve herinnering stellen.

## Thema’s en vragen

### De kindertijd (0-12 jaar)

**Doel**

Het trainen van de patiënt in het ophalen van specifieke, positieve herinneringen. De periode in dit gesprek is de kindertijd tot 12 jaar.

**Opbouw** (is steeds gelijk in gesprekken 2, 3 en 4):
1) een korte inleiding en herhaling van de rationale,

2) – eventueel – een korte bespreking van een huiswerkopdracht,

3) de eigenlijke training in het ophalen van de herinneringen (8 á 10 vragen)

4) evaluatie en feedback,

5) bespreking van een nieuwe huiswerkopdracht (wanneer van toepassing) en planning van een volgend gesprek.

**Introductie**

‘Vandaag wil ik het met u hebben over uw kindertijd. De periode tot uw 12^e^ jaar. Het is dus de periode dat u op de lagere school zat.

Ik ben vooral heel benieuwd naar specifieke gebeurtenissen in deze periode die voor u bijzonder waren. Het gaat mij dus om momenten die eenmalig waren.

U heeft me de vorige keer al iets over uw kindertijd verteld, daar wil ik u nog wat meer over vragen.’

*Loop kort met de patiënt na wat zij/hij je verteld heeft over de kindertijd in het oriënterende gesprek en stel wat aanvullende vragen of vraag de patiënt om over bepaalde onderwerpen wat meer te vertellen. Wanneer de patiënt goed op de kindertijd gefocust is, kun je overgaan tot het stellen van vragen over specifieke herinneringen:*

‘Ik heb nu een beeld gekregen van uw kindertijd. Nu stel ik u een aantal vragen over specifieke gebeurtenissen in uw kindertijd.’

**Thema’s en vragen**

**Startvragen**

1. Herinnert u zich een favoriet of speciaal speelgoed of spelletje dat u vaak deed en u veel plezier gaf?
2. Had u een juf of meester waar u dol op was? Wat vond u zo geweldig aan hem of haar?
3. Had u een vriendje of vriendinnetje (buurjongen –meisje) waar u veel mee speelde? Kunt u zich een leuk moment herinneren toen u met hem/haar speelde, toen u bijvoorbeeld leuk kattenkwaad hebt uitgehaald?

**Overige vragen**

1. Kunt u me vertellen over een uitje dat u met uw ouders (of een andere dierbare) heeft gemaakt? (eventueel specifiek maken: uitje naar het strand, de kermis, carnaval etc.)
2. Kunt u zich een leuk moment herinneren dat u had met één van uw broers of zussen, toen u jong was? (Als er geen broers of zussen waren, dan een dierbaar iemand).
3. Kunt u zich een verjaardag herinneren uit die tijd die voor u speciaal was? Wie waren er en wat gebeurde er?
4. Kunt u zich een leuk sinterklaasfeest/ een leuke feestdag herinneren?
5. Met wie kon u het goed vinden toen u op de lagere school zat?
6. Kunt u zich een moment op de lagere school herinneren waarin u zich heel blij voelde?
7. Heeft u, toen u zo klein was, wel eens een cadeau ontvangen waar u heel erg blij mee was? Kunt u me daar wat meer over vertellen?
8. Kunt u zich een zeer gelukkige dag herinneren die u als kind samen met uw ouders beleefde?
9. Kunt u me een voorval vertellen waarbij uw vader of moeder iets deed toen u nog een kind was en dat u heel blij of gelukkig maakte?
10. Herinnert u zich dat u een keer iets bijzonders heeft gedaan met een vriendje of vriendinnetje, wat u bijstaat als een heerlijke tijd? Waarom was deze dag zo speciaal voor u als kind?
11. Herinnert u zich een moment uit uw jeugd dat u zich geliefd of ondersteund voelde door iemand?
12. Kunt u een voorbeeld noemen van een dag of voorval dat uw vader of moeder iets deed waardoor u heel trots op hem of haar was en u erg van hem of haar hield?
13. Herinnert u zich een dag waarop u een kus of een knuffel kreeg van iemand omdat u iets had gedaan?

### De jeugd (12 – 18 jaar)

**Doel** :

Het trainen van de patiënt in het ophalen van specifieke, positieve herinneringen. De periode in dit gesprek is de jeugd, van 12 tot 18 jaar.

**Opbouw:**1) een korte inleiding en herhaling van de rationale,

2) – eventueel – een korte bespreking van een huiswerkopdracht,

3) eigenlijke training in ophalen van de herinneringen (8 á 10 vragen)

4) evaluatie en feedback,

5) bespreking van een nieuwe huiswerkopdracht (wanneer van toepassing) en planning van een volgend gesprek.

**Introductie**

‘Als u het goed vindt, wil ik nu graag stil staan bij uw herinneringen aan de tijd dat u ongeveer 12 tot 18 jaar oud was. Ik zal u vragen stellen over specifieke gebeurtenissen die bijzonder waren en die niet terugkwamen. Gebeurtenissen die u heel goed zijn bijgebleven en die u als het ware nog helemaal voor de geest staan. Het eerste dat bij u opkomt is goed. Maar zullen we nog even terugkomen op wat u mij al verteld heeft over uw jeugd?’

*Loop kort met de patiënt na wat hij/zij je verteld heeft over zijn/haar jeugd in het oriënterende gesprek en stel wat aanvullende vragen of vraag de patiënt om over bepaalde onderwerpen wat meer te vertellen. Wanneer de patiënt goed op zijn/haar jeugd gefocust is, kun je overgaan tot het stellen van vragen over specifieke herinneringen:*

‘Ik heb nu een beeld gekregen van uw jeugd. Nu stel ik u een aantal vragen over specifieke gebeurtenissen in uw jeugd. Zullen we beginnen?’

**Thema’s en mogelijke vragen**

**Startvragen**

1. Kunt u zich een verjaardag herinneren uit die tijd die voor u speciaal was? Wie waren er en wat gebeurde er?
2. Herinnert u zich nog een moment als tiener dat u heel enthousiast was over een boek, een film, een persoon of iets anders?
3. Herinnert u zich een dag of een viering op school of uw werk waarbij iedereen veel plezier had? Of is er een ander feest of feestdag geweest die u is bijgebleven?

**Overige vragen**

1. Herinnert u zich een speciaal moment dat u bijvoorbeeld uw eerste kus kreeg of dat u verliefd was op iemand? [realiseer dat deze vraag bij sommige mensen wat ongemak kan veroorzaken]
2. Is er een moment geweest tussen uw 12de en 18de levensjaar dat u iets bijzonders deed in de kerk, op school of een sportvereniging, dat u altijd is bijgebleven?
3. Herinnert u zich een plezierige dag of moment dat u met uw ouders er op uit trok?
4. Herinnert u zich een dag, tussen uw 12^de^ en 18^de^, dat u zich heel gelukkig voelde? Een bijzondere dag waar u later nog vaak aan moest denken? Wat gebeurde toen?
5. Is er wel eens een dag geweest tussen uw 12^e^ en 18^e^ dat u uw ouders heeft geholpen met een moeilijke klus en dat het u veel voldoening gaf toen deze klus was geklaard?
6. Had u in uw tienertijd een speciale vriend/vriendin? Waarom was hij/zij zo speciaal voor u? Heeft u met hem/haar iets bijzonders doorgemaakt? (dorvragen en specifiek maken)
7. Vertelt u me eens over een speciale dag, toen u tussen de 12 en 18 was, die u doorbracht met één van uw beste vrienden/vriendinnen (evt. broers of zussen).
8. Herinnert u zich een moment tussen uw 12^de^ en 18^de^, dat u zich beschermd of geliefd voelde terwijl u bij iemand was?
9. Wat is de plezierigste herinnering die u heeft aan uw grootouders toen u tussen de 12 en 18 was? (Indien geen grootouders, dan andere familie).
10. Wanneer u al deze vragen beschouwt, welk moment was voor u het gelukkigst? Kunt u dat uitleggen?

### De volwassenheid

**Doel**:

Het trainen van de patiënt in het ophalen van specifieke, positieve herinneringen. De periode in deze gesprek is *de volwassenheid*. Deze periode kan verdeeld worden over twee gesprekken: de eerste over de volwassenheid tot het 45^ste^ levensjaar, de tweede over de jaren daarna.

**Opbouw**:
1) een korte inleiding en herhaling van de rationale,

2) – eventueel – een korte bespreking van een huiswerkopdracht,

3) eigenlijke training in ophalen van de herinneringen (8 á 10 vragen)

4) evaluatie en feedback,

5) bespreking van een nieuwe huiswerkopdracht (wanneer van toepassing) en planning van een volgende gesprek.

**Introductie van training binnen het gesprek**

*De volwassenheid beslaat een grote periode en er zijn veel vragen mogelijk. Welke vragen relevant zijn, is erg afhankelijk van de leeftijd en het leven van de patiënt. Het oriënterende gesprek zal daarvoor richting hebben gegeven. We geven veel vragen weer, waaruit je een keuze kunt maken. Je kunt dit ook in overleg doen met de patiënt.*

‘Als u het goed vindt, wil ik nu graag stil staan bij uw herinneringen aan de tijd dat u volwassen was. Ik zal u vragen stellen over specifieke gebeurtenissen die bijzonder waren en die niet terugkwamen. Gebeurtenissen die u heel goed zijn bijgebleven en die u als het ware nog helemaal voor de geest staan. Het eerste dat bij u opkomt is goed.

Maar zullen we nog even terugkomen op wat u mij al verteld heeft over uw volwassen leven?’

*Loop kort met de patiënt na wat hij/zij je verteld heeft over zijn/haar volwassen leven in het oriënterende gesprek en stel wat aanvullende vragen of vraag de patiënt om over bepaalde onderwerpen wat meer te vertellen. Wanneer de patiënt goed op zijn/haar volwassenheid gefocust is, kun je op basis van het oriënterende (eerste) gesprek zelf kiezen voor een periode die veel mogelijkheden biedt voor het ophalen van specifieke positieve herinneringen of dit met de patiënt bespreken:*

‘Ik heb nu een beeld gekregen van uw leven.

Als het gaat om uw volwassen leven, wat was voor u de beste periode?

Hoe kwam dat zo?

Kunt u daar een voorbeeld van geven?’

*En dan doorgaan met vragen naar specifieke positieve gebeurtenissen in de betreffende periode.*

‘Als u het goed vindt, wil ik nu graag stil staan bij uw herinneringen aan de tijd dat u volwassen was. Ik zal u vragen stellen over specifieke gebeurtenissen die bijzonder waren en die niet terugkwamen. Gebeurtenissen die u heel goed zijn bijgebleven en die u als het ware nog helemaal voor de geest staan. Het eerste dat bij u opkomt is goed. Zullen we beginnen?’

**Thema’s en mogelijke vragen**

**Startvragen**

1. Is er een bepaalde plaats of plek die veel voor u betekende in de periode van uw 18^de^ tot ongeveer uw 45^ste^ levensjaar? Aan welke gebeurtenis of moment is die betekenis of plek gekoppeld? Hoe zag deze plek eruit?
2. Kunt u iets vertellen over een belangrijke relatie in uw leven? Hoe heeft u deze persoon leren kennen? Kun u mij een of enkele momenten noemen die uw relatie dierbaar voor u maken? Wat gebeurde er toen? (Verder concretiseren, hoe oud was toen etc.) Wat was een moment in deze relatie dat u heel erg dierbaar is?
3. Wat is een heel belangrijke positieve gebeurtenis geweest voor u na uw 18^e^ levensjaar? (eventueel doorvragen, wanneer was dit, hoe oud was u toen etc. concreet maken)

**Overige vragen**

1. Kunt u zich een dag herinneren dat u iets moest bereiken (organiseren, prestatie etc.) waarin u ook slaagde en waardoor u zich erg trots op uzelf voelde?
2. Herinnert u zich een moment dat u deelnam aan een team of groep (kerk, sport, natuur) dat u erg gelukkig was?
3. Kunt u nog een bijzondere gebeurtenis noemen die er voor u uit springt tussen uw 20^ste^ en 40^ste^ levensjaar? (eventueel doorvragen, wanneer was dit, hoe oud was u toen etc. concreet maken)
4. Op welke wijze maakte u plezier of zocht u ontspanning? Kunt u zich daarbij nog een moment herinneren die als voorbeeld kan dienen?
5. Wat was een moment in uw dagelijkse bezigheden dat u heel erg trots was op u zelf?
6. Kunt u zich een moment herinneren uit de periode van tussen de 18de en de 45ste levensjaar dat u zich heel erg geliefd voelde? Of verrast? Of ontroerd?
7. [een vraag over kinderen] Herinnert u zich de geboorte van uw dochter/zoon (*specifiek maken*)? Kunt u een leuk grappig voorval herinneren toen hij/zij klein was? Wat was een moment dat u heel erg trots op hem/haar was?
8. Is er moment geweest dat u moest opkomen voor u zelf of een dierbare en dat ook lukte?
9. Is er een periode geweest die veel van u vroeg, maar dat u er uiteindelijk goed door heen bent gekomen? Herinnert u zich nog het moment dat u besefte dat het goed was gekomen?
10. Herinnert u zich een dag dat u iets bijzonders heeft gedaan voor iemand en dat u dat erg gelukkig maakte?

**Vragen die meer gaan over tweede levenshelft**

**Startvragen**

1. Heeft u in deze periode een reis/vakantie gemaakt die u nooit meer zal vergeten? Wat was een bijzonder moment tijdens deze vakantie/reis?
2. Vertelt u eens over een mooi moment uit uw huwelijk? Bijvoorbeeld een reis die samen maakte of een dat samen iets bereikte waar u heel trots op was.
3. In deze periode, wie was de meest belangrijkste persoon voor u? Waarom? Kunt u over een mooie gebeurtenis met hem/haar vertellen?

**Overige vragen**

1. Kunt u zich iets herinneren over uw kinderen toen ze al wat groter waren, dat u heel erg trots op ze was? (specifiek maken: waar was u zo trots op, etc.?)
2. Vertelt u eens over goede vrienden waar mee u een langdurige mooie vriendschap had tijdens uw leven. Was er iets wat u speciaal met deze vriend/-in deed waar u met plezier aan kunt denken?
3. Welke bezigheden had u buiten het werk waar u een goed gevoel over had? Vertelt u daar eens wat meer over, wat vond u er zo leuk aan?
4. Wat staat u bij uit deze periode, zeg maar een levenswijsheid die u graag door zou willen geven aan de volgende generatie.
5. Heeft u kleinkinderen? Wat doet of deed u zoal met hen? Kunt u zich een dag herinneren dat u met uw kleinkinderen iets gedaan heeft, een hele speciale dag?
6. Herinnert u zich een voorval van uw werk, dat u iets moest doen of regelen, waar u nog steeds heel trots op bent?
7. Herinnert u zich nog een publiek persoon uit die tijd die veel indruk op u heeft gemaakt?
8. Herinnert u zich een dag dat u iets bijzonders heeft gedaan voor iemand en dat u dat erg gelukkig maakte?

### Het leven in het algemeen

**Doel**:

Het trainen van de patiënt om specifieke, positieve herinneringen op te halen en het afsluiten van de therapie.

**Opbouw**:
1) korte inleiding en uitleg doel van het laatste gesprek

2) terugkijken naar de vorige gesprekken, algemene feedback,

3) training met vragen over het leven in het algemeen (5 á 8) en feedback;

4) afbouwen therapie en afspraken over mogelijke zelfstandige stappen door de patiënt;

5) afspraken over nameting/ eventueel mogelijkheden voor andere vormen van nazorg.

In dit gesprek bespreek je kort wat het doel is van de laatste bijeenkomst, kijk je samen naar de vorige gesprekken, oefen je nog met een paar vragen over het leven in het algemeen en sluit de therapie af met een algemene evaluatie. Indien mogelijk maak je afspraken over oefeningen die de patiënt zelf kan doen.

N.B. een week na het laatste gesprek zal de onderzoeker de nameting verrichten. Mocht daaruit naar voren komen dat er nog steeds sprake is van een verhoogd level van depressieve klachten dan kan deze met de patiënt bespreken op welke manier de patiënt verdere nazorg kan krijgen.

**Introductie**

*De vragen over het leven in het algemeen komen in het laatste gesprek aan de orde. Nu is het niet nodig om iemands geheugen te richten op een specifieke periode en kun je direct beginnen met de vragen.*

‘Als u het goed vindt, wil ik nu graag stil staan bij uw herinneringen aan uw leven in het algemeen. Ik zal u vragen stellen over specifieke gebeurtenissen die belangrijk voor u zijn. Gebeurtenissen die u heel goed zijn bijgebleven en die u als het ware nog helemaal voor de geest staan. Het eerste dat bij u opkomt is goed. Zullen we beginnen?’

**Thema’s en vragen**

**Startvraag (zie ook afsluitende vragen)**

1. Stel u mag een moment uit uw leven nog een keer meemaken. Welk moment uit uw leven zou u dan kiezen?

**Afsluitende vragen**

1. Laten we het nog even hebben over uw leven nu, wat vindt u het fijnste aan uw leven zoals het nu is?
2. Aan welke gebeurtenis in uw recente verleden (de laatste jaren) heeft u een fijne herinnering?
3. Wat hoopt u nog mee te maken in de komende tijd?

**Overige vragen**

1. Wat is een heel gelukkig moment geweest in uw leven?
2. Wat is een belangrijke positieve les die het leven u heeft gegeven? Weet u nog wanneer u dit besefte?
3. Wat is de mooiste herinnering aan een moment in uw leven?
4. Wat was de gelukkigste periode in uw leven? Welke gebeurtenis luidde deze periode in? Welk moment symboliseert deze periode het meest?
5. Welk moment in uw leven is voor u verbonden met de meeste fijne emoties?
6. Wat ziet u nu als het belangrijkste positieve keerpunt in uw leven?
7. Wanneer was u het meest trots op u zelf in u leven?
8. Wanneer was u het meest trots op een dierbare ander in uw leven?
9. Wie bent u voor altijd dankbaar omdat hij of zij iets belangrijks voor u heeft gedaan? Aan welke gebeurtenis/moment is dat vooral verbonden?
10. Als u uw hele leven dezelfde leeftijd zou kunnen houden, welke leeftijd zou dat dan zijn?

2. Aandachtspunten bij de uitvoering

**Stel jezelf voortdurend de vraag: ‘vertelt de ander mij nu een specifieke, positieve herinnering die hem/haar bekrachtigt?’.**

Het belangrijkste is om steeds als begeleider na te gaan of de verhalen die de patiënt vertelt **specifiek** en **positief** zijn, dat wil zeggen bijdragen aan de doelstelling van de gesprekken. Let goed op non-verbale gedragingen (lichaamsgedrag, gezichtsexpressie) die zichtbaar maken of het onderwerp de ander ‘goed’ doet. Zo niet, probeer het gesprek weer naar specifieke, positieve ervaringen te leiden.

**Neem de tijd om door te vragen.**

- Hoe ging dat dan precies?
- Wat deed u (de ander) toen?
- Hoe zag de omgeving er precies uit?
- Weet u nog hoe u zich toen voelde?
- Wat deed u toen precies?

Liever zes vragen goed behandeld dan alle vragen afgeraffeld. Ga aan de andere kant door met een volgende vraag wanneer je merkt dat een vraag weinig oproept. Het kan ook goed zijn om de patiënt te vragen of hij/zij het een leuke of geen leuke vraag vindt.

In het begin zullen sommige deelnemers moeite hebben om zich details weer te herinneren. Neem de tijd om de herinnering in al zijn facetten te verkennen. Neem vooral de tijd voor herinneringen waarvan duidelijk is dat zij veel (positiefs) voor hem/haar betekenen. Als het goed gaat hoef je in de loop van de gesprekken steeds minder door te vragen en kun je steeds meer een stapje terug doen.

De kunst is om de balans te vinden tussen doorvragen en niet teveel aandringen. Juist in het begin kost het de patiënt veel moeite om zich specifieke positieve gebeurtenissen te herinneren. Het vraagt geduld en doorzettingsvermogen om de patiënt toch op dat spoor te zetten. Zoek naar een thema dat toegang geeft tot specifieke herinneringen. Probeer daarbij de patiënt niet te frustreren, en voorkom dat ze het gevoel krijgen niet te kunnen voldoen aan wat er wordt gevraagd. In dat geval kan het beter zijn om wat meer ruimte te nemen voor algemene herinneringen en dan gaandeweg te zoeken naar specifieke momenten. Het is echter niet de bedoeling te lang op het algemene niveau te blijven. De interventie zal dan niet werken.

**Stel je empathisch en begripvol op**

Wees belangstellend en leef je in, maar let op dat het hoofddoel van een gesprek – het leren om specifieke, positieve herinneringen op te halen – niet verloren gaat. Met andere woorden: het gaat niet om het levensverhaal, maar om de training. Dit betekent dat je actief en sturend moet zijn –in plaats van volgend- en uitweidingen moet inperken. Ook moet je loslaten inzichtgevend te willen zijn.

**Wees je bewust van de betekenis van de gesprekken**

Dit lijkt misschien vanzelfsprekend, maar het is belangrijk om je te realiseren dat je nogal iets van mensen vraagt. In een aantal gesprekken staan zij stil bij belangrijke ervaringen in hun leven en maken zij ‘de balans op’. Dit kan zeer emotioneel voor mensen zijn.

**Wees genereus met complimenten maar wel gemeend.**

Complimenten kunnen zowel gegeven worden naar aanleiding van het vertelde ‘Dat is wel heel moedig van u geweest!’ als het proces van vertellen ‘ik vind dat u zich nog veel kan herinneren’. Maar niets is zo dodelijk als een ‘onecht’ compliment.

Nog mooier is het wanneer je de deelnemers kunt verleiden om zichzelf complimenten te geven. Dit werkt krachtiger. Dit is mogelijk door bijvoorbeeld te vragen: wat vindt u er nu van dat u dat toen zo deed? Wat zeiden anderen in uw omgeving over uw prestatie?

**Geef regelmatig gevoelsreflecties**

Een gevoelsreflectie is een kort antwoord waarbij de je de kern van de beleving terugkoppelt. ‘Dat moet wel heel spannend voor u zijn geweest’, ‘voelde dat voor u als een overwinning?’. Dit kan de evaluatieve afsluiting van een vraag zijn, waarin subtiel het positieve, bekrachtigende van de vertelde ervaring wordt uitgelicht.

**Wees voorzichtig met algemene evaluatieve opmerkingen als ‘u heeft toch wel een mooi leven gehad’.**

Want er bestaat dan de kans dat de patiënt gaat tegensputteren en juist de negatieve evaluatie gaat belichten. Geef iemand de tijd om zelf het beeld van zijn leven bij stellen. Vertrouw op het proces en ga niet te veel ‘trekken’.

**Breng iemand vanuit begrip weer terug naar het onderwerp**

Als je merkt dat iemand afdwaalt, klaagt, of vooral op negatieve ervaringen focust, probeer dan iemand op een begripsvolle wijze weer naar de doelstelling van de gesprekken te leiden. Dein mee en neutraliseer. ‘Ik begrijp dat dit voor u heel moeilijk is geweest maar zou u nog iets meer kunnen vertellen over…’.

**Houd de spanningsboog van de patiënt goed in de gaten.**

De richtlijn voor een gesprek is 60-90 minuten. Dit is redelijk lang. Vooral wanneer er emotionele onderwerpen aan de orde zijn geweest. Let daarom goed op signalen van vermoeidheid bij de patiënt. Aarzel niet om zo nodig eerder te stoppen.

**Omgaan met emoties**

Het is zeer goed mogelijk dat patiënten tijdens de gesprekken emotioneel worden. De herinneringen kunnen veel oproepen. Geef iemand de tijd om te herstellen. ‘Dit onderwerp raakt u erg’. ‘Neemt u maar even de tijd’. Aanwezig zijn is op deze momenten het belangrijkst. Probeer niet vanuit onmacht te gaan ‘redderen’. De meeste mensen kunnen na een paar minuten de draad van het gesprek weer oppakken. Je kunt de vraag stellen of ‘de ander juist nog even over door wil praten of liever op een ander onderwerp doorgaat’.

**Appendix B: Algemene vragen (demografie; 12 items)**

1. Wat is uw geboortedatum: _ _ - _ _ - _ _ _ _

2. Geslacht respondent:

- man
- vrouw

3. In welk land bent u geboren?

- Nederland
- Marokko
- Turkije
- Suriname
- ander land, namelijk _______________________________

4. Wat is uw burgerlijke staat?

- Gehuwd
- Gescheiden
- Verweduwd, in ….. (jaartal invullen)
- Nooit gehuwd geweest

5. Heeft u kinderen?

- Nee
- Ja, namelijk ….. (aantal invullen)

6. Wat is de hoogste school of opleiding die u heeft voltooid?

- Geen
- Lagere school
- Lager beroepsonderwijs (bijv. LEAO, LHNO, LTS)
- Middelbaar algemeen onderwijs (bijv. MAVO, (M)ULO, IVO, LAVO, 3-jarige HBS)
- Middelbaar beroepsonderwijs (bijv. MEAO, MTS, INAS)
- Hoger algemeen onderwijs (bijv HAVO, VWO, 5-jarige HBS, MMS, Atheneum, Gymnasium)
- Hoger beroepsonderwijs (bijv. HEAO, HTS, MO-A, MO-B, SA, PA)
- Academisch onderwijs (Universiteit, TH)
- Anders, namelijk __________________________________

7. Bent u lid van een kerk of godsdienstige groepering?

- Nee
- Nederlands hervormd
- Gereformeerd
- Rooms-katholiek
- Overig christelijk
- Joods
- Islamitisch
- Hindoeïstisch
- Humanistisch
- Anders

**Appendix C: EORTC-QLQ-PAL-15**

Kwaliteit van Leven

Wij zijn geïnteresseerd in bepaalde dingen over u en uw gezondheid. Wilt u alle

vragen zelf beantwoorden door het getal te omcirkelen dat het meest op u van

toepassing is.

Er zijn geen "juiste" of "onjuiste" antwoorden. De informatie die u geeft

zal strikt vertrouwelijk worden behandeld.

De antwoordmogelijkheden zijn:

1 =Helemaal niet; 2 = Een beetje, 3 = Nogal; 4 = Heel erg

|  | 1 | 2 | 3 | 4 |
| --- | --- | --- | --- | --- |
| 1. Heeft u moeite met het maken van een korte wandeling buitenshuis? |  |  |  |  |
| 2.. Moet u overdag in bed of op een stoel blijven? |  |  |  |  |
| 3. Heeft u hulp nodig met eten, aankleden, uzelf  wassen of naar het toilet gaan? |  |  |  |  |
| 4. Was u kortademig? |  |  |  |  |
| 5. Heeft u pijn gehad? |  |  |  |  |
| 6. Heeft u moeite met slapen gehad? |  |  |  |  |
| 7. Heeft u zich slap gevoeld? |  |  |  |  |
| 8. Heeft u gebrek aan eetlust gehad? |  |  |  |  |
| 9. Heeft u zich misselijk gevoeld? |  |  |  |  |
| Gedurende de afgelopen week: |  |  |  |  |
| 10. Had u last van obstipatie? (was u verstopt?) |  |  |  |  |
| 11. Was u moe? |  |  |  |  |
| 12. Heeft pijn u gehinderd in uw dagelijkse bezigheden? |  |  |  |  |
| 13. Voelde u zich gespannen? |  |  |  |  |
| 14. Voelde u zich neerslachtig? |  |  |  |  |

Wilt u voor de volgende vraag het getal tussen 1 en 7 omcirkelen dat het

meest op u van toepassing is

15. Hoe zou u uw algehele "kwaliteit van het leven" gedurende de afgelopen week

beoordelen?

1 2 3 4 5 6 7

**Appendix D: AMT**

**Autobiografische Geheugenlijst**

Dit onderdeel gaat over herinneringen aan gebeurtenissen die u zelf heeft meegemaakt.

Ik zal u uitleggen wat de bedoeling is. Ik heb hier 10 kaarten. Op deze 10 kaarten staan woorden. Ik wil u zo dadelijk vragen deze woorden te lezen. U wordt gevraagd een moment te herinneren dat u het woord dat op het kaartje staat, vertoonde.

De gebeurtenis kan pas geleden plaats hebben gevonden (bijvoorbeeld gisteren of in de afgelopen week), maar ook heel lang geleden. Het kan om een heel belangrijke gebeurtenis gaan, maar ook om iets kleins. Het is belangrijk dat de herinneringen die u noemt specifiek zijn. Daarmee bedoel ik dat de herinneringen verwijzen naar een zekere gebeurtenis die op een bepaalde dag plaatsvond.

Ik zal u een voorbeeld van een specifieke herinnering geven: op dit kaartje staat het woord 'creatief'. BLAUWE KAART MET 'CREATIEF' LATEN ZIEN. Stel dat u als herinnering noemt: "ik ben altijd al creatief geweest ". Een dergelijke uitspraak verwijst niet naar een bepaalde gebeurtenis, maar naar een heleboel gebeurtenissen. Als u zegt: "ik vond mezelf heel creatief toen ik mijn eerste schilderij afhad" dan is dat wel een specifieke gebeurtenis.

VRAAG VOOR IEDER WOORD:

**Kunt u zich een specifiek moment herinneringen dat u …………… was?**

LEES DE WOORDEN HARDOP VOOR, TEGELIJK MET HET TONEN VAN DE KAART.

ALS DE PERSOON EEN ALGEMENE HERINNERING NOEMT, ZEG DAN:

**Kunt u zich een specifiek moment, één bepaalde gebeurtenis herinneren?**

DOE DIT MAXIMAAL EENMAAL PER WOORD.

VRAAG NA IEDERE SPECIFIEKE HERINNERING:

**wanneer vond deze gebeurtenis plaats?**

HERHAAL DE INSTRUCTIE ALS DE INSTRUCTIE NIET BEGREPEN WORDT.

AMT – pre measurement

1 = specifieke herinnering

2 = specifieke herinnering na 1x doorvragen

3 = categoric/generic

4 = extended

5 = voldoet niet aan de opdracht

9 = geen herinnering (geen antwoord); 8 = per ongeluk overgeslagen (of onverstaanbaar)

Extra: dus altijd in combinatie met een scoringscategorie: S = indien zelfde herinnering (S van same); Nd = niet doorgevraagd

| VERSIE 1 | Tekening | **Bang** | **Succes** | **Fiets** | **Restaurant** | **Reis** | **Optreden** | **Boos** | **School** | **Dier** |
| --- | --- | --- | --- | --- | --- | --- | --- | --- | --- | --- |
| Code |  |  |  |  |  |  |  |  |  |  |
| Periode |  |  |  |  |  |  |  |  |  |  |
| Seconden |  |  |  |  |  |  |  |  |  |  |
|  |  |  |  |  |  |  |  |  |  |  |
|  |  |  |  |  |  |  |  |  |  |  |
|  |  |  |  |  |  |  |  |  |  |  |

AMT - post measurement

1 = specifieke herinnering

2 = specifieke herinnering na 1x doorvragen

3 = categoric/generic

4 = extended

5 = voldoet niet aan de opdracht

9 = geen herinnering (geen antwoord); 8 = per ongeluk overgeslagen (of onverstaanbaar)

Extra: dus altijd in combinatie met een scoringscategorie: S = indien zelfde herinnering (S van same); Nd = niet doorgevraagd

| VERSIE 2 | Cadeau | **Televisie** | **Saai** | **Trein** | **Enthousiast** | **Telefoon** | **Opdracht** | **Teleurgesteld** | **Grappig** | **Uitstapje** |
| --- | --- | --- | --- | --- | --- | --- | --- | --- | --- | --- |
| Code |  |  |  |  |  |  |  |  |  |  |
| Periode |  |  |  |  |  |  |  |  |  |  |
| Seconden |  |  |  |  |  |  |  |  |  |  |
|  |  |  |  |  |  |  |  |  |  |  |
|  |  |  |  |  |  |  |  |  |  |  |
|  |  |  |  |  |  |  |  |  |  |  |

**Appendix E: NEIS**

**Levenservaring**

Bij de volgende vragen is het de bedoeling dat u weer aangeeft in welke mate u het eens bent met de stellingen.

|  | **Helemaal oneens** | **Oneens** | **Enigszins oneens** | **Enigszins eens** | **Eens** | **Helemaal eens** |
| --- | --- | --- | --- | --- | --- | --- |
| Ik besef de waarde van al mijn ervaringen. | 0 | 0 | 0 | 0 | 0 | 0 |
| Het doet me pijn om na te denken over de dromen en doelen die ik had maar niet hebt vervuld. | 0 | 0 | 0 | 0 | 0 | 0 |
| Ik heb alles wat ik wilde volbracht in mijn leven. | 0 | 0 | 0 | 0 | 0 | 0 |
| Het leven is zinvol. | 0 | 0 | 0 | 0 | 0 | 0 |
| Ik zou willen dat ik meer lief had gehad in mijn leven. | 0 | 0 | 0 | 0 | 0 | 0 |
| Ik ben nu op een punt aangekomen waarop ik kan accepteren dat de gebeurtenissen in mijn leven nodig waren. | 0 | 0 | 0 | 0 | 0 | 0 |
| Naarmate ik ouder word, begrijp ik mensen beter. | 0 | 0 | 0 | 0 | 0 | 0 |
| Ik heb last van de fouten die ik in het verleden heb gemaakt. | 0 | 0 | 0 | 0 | 0 | 0 |
| Ik heb precies gedaan wat ik wilde met mijn leven. | 0 | 0 | 0 | 0 | 0 | 0 |
| Ik zie een betekenisvolle rode draad door de vele gebeurtenissen in mijn leven lopen. | 0 | 0 | 0 | 0 | 0 | 0 |
| Wanneer ik geboren zou zijn met een andere persoonlijkheid, zou het leven beter zijn geweest. | 0 | 0 | 0 | 0 | 0 | 0 |
| Ik kan de dood waardig onder ogen zien. | 0 | 0 | 0 | 0 | 0 | 0 |
| Zelfs mijn momenten van lijden hebben betekenis gehad. | 0 | 0 | 0 | 0 | 0 | 0 |
| Ik zou willen dat ik meer tijd had om een andere weg in te slaan in mijn leven. | 0 | 0 | 0 | 0 | 0 | 0 |
| Naarmate ik ouder word, begrijp ik mijn levensverhaal beter. | 0 | 0 | 0 | 0 | 0 | 0 |

**Appendix F: HADS**

**Appendix G: MINI**

| **Depressieve Episode** | |  |  |  |  |
| --- | --- | --- | --- | --- | --- |
| **A1** | a. Hebt u zich ooit, gedurende tenminste 2 weken, voortdurend depressief of neerslachtig gevoeld, tijdens het grootste gedeelte van de dag, en dit bijna elke dag? | NEE | JA |  |  |
|  |  |  |  |  |  |
|  | b. Voelt u zich voortdurend depressief of neerslachtig tijdens het grootste deel van de dag, bijna elke dag gedurende de afgelopen 2 weken? | NEE | JA |  |  |
|  |  |  |  |  |  |
| **A2** | a. Hebt u ooit, gedurende tenminste 2 weken, minder interesse, minder zin en plezier gehad in de dingen die u tevoren wel aanspraken? | NEE | JA |  |  |
|  |  |  |  |  |  |
|  | b. Had u gedurende de afgelopen 2 weken minder interesse of minder plezier in de dingen die u tevoren wel aanspraken? | NEE | JA |  |  |
|  |  |  |  |  |  |
|  | **IS OP A1a, A1b, A2a en op A2b NEE GEANTWOORD, GA DAN NAAR VOLGENDE SECTIE. GA ANDERS VERDER MET DE VOLGENDE VRAGEN (VOOR HUIDIGE EPISODE EN/OF EPISODE IN HET VERLEDEN)** |  |  |  |  |
|  |  |  |  |  |  |
|  | **INDIEN MOMENTEEL DEPRESSIEF (A1b OF A2b= JA): ONDERZOEK ALLEEN DAN EERST DE HUIDIGE EPISODE, EN DAARNA DE EPISODE IN HET VERLEDEN.**  **INDIEN NEE: ONDERZOEK DE MEEST SYMPTOMATISCHE EPISODE IN HET VERLEDEN.** |  |  |  |  |
|  |  |  |  |  |  |
| **A3** | **In de periode van 2 weken waarin u zich depressief of**  **minder geïnteresseerd voelde:** |  | |  | |
|  |  | Huidige Episode | | Verleden | |
|  | a. Had u bijna elke dag minder of meer eetlust? **OF** | NEE | JA | NEE | JA |
|  | Is uw gewicht af- of toegenomen zonder dat u dat wilde? (BV. MET ± 5% VAN HET LICHAAMSGEWICHT OF MET ONGEVEER 3½ KG VOOR EEN PERSOON VAN ONGEVEER 70 KG IN EEN MAAND). INDIEN EEN VAN BEIDE = JA: CODEER JA |  |  |  |  |
|  | b. Had u bijna elke nacht slaapproblemen (inslaapmoeilijkheden, wakker worden ’s nachts, voortijdig ontwaken of overmatig slapen)? | NEE | JA | NEE | JA |
|  | c. Sprak of bewoog u trager dan anders, of was u zenuwachtig, rusteloos of had u moeite met stilzitten, en dat bijna iedere dag? | NEE | JA | NEE | JA |
|  | d. Voelde u zich bijna iedere dag moe en futloos? | NEE | JA | NEE | JA |
|  |  |  |  |  |  |
|  | e. Voelde u zich bijna iedere dag waardeloos of schuldig? | NEE | JA | NEE | JA |
|  |  |  |  |  |  |
|  | f. Had u bijna iedere dag moeite met concentreren of beslissingen nemen? | NEE | JA | NEE | JA |
|  | g. Had u herhaaldelijk gedachtes aan de dood? En wenste u dat u dood was? Overwoog u uzelf letsel toe te brengen, uzelf iets aan te doen? | NEE | JA | NEE | JA |
| **A4** | GA VOOR DE PERIODE IN HET VERLEDEN NA OF DE AANWEZIGE SYMPTOMEN IN DEZELFDE PERIODE VAN TWEE WEKEN AANWEZIG WAREN |  |  | NEE | JA |
|  |  |  |  |  |  |
| **A5** | Verstoorden de depressieve symptomen in belangrijke mate uw algemeen dagelijks functioneren, zoals verzorgingstaken en de omgang met anderen, of het functioneren op belangrijke andere terreinen? | NEE | JA | NEE | JA |
|  |  |  |  |  |  |
|  |  |  |  |  |  |
|  | CHRONOLOGIE |  | | | |
| **A11** | Hoe oud was u toen de eerste verschijnselen van depressie zich voordeden? |  | | | |
|  |  |  |  |  |  |
| **A12** | Hoeveel periodes hebt u zich in uw leven depressief gevoeld en deze verschijnselen gehad (dagelijks gedurende tenminste 2 weken)? | Aantal episodes: ………… | | | |
|  |  |  | | | |

**Appendix H: TIC-p**

**Vragenlijst ziekte en zorggebruik**

**A. Zorggebruik**

De volgende vragen gaan over zorggebruik. Wij zijn geïnteres­seerd in het aantal keren dat u in de afgelo­pen **4 weken** gebruik heeft gemaakt van de voorzieningen in de gezondheidszorg.

|  | **Heeft u in de afgelopen 4 weken contact gehad met:** | **nee** | **Zo ja,**  **hoeveel keer?** |
| --- | --- | --- | --- |
| A1 | Huisarts | 🞎 | ____ keer |
| A2. | Bedrijfsarts of ARBO arts | 🞎 | ____ keer |
| A3. | Bedrijfsmaatschappelijk werker | 🞎 | ____ keer |
| A4. | Maatschappelijk werker (denk ook aan: MEE Amstel en Zaan; Breed Platform Verzekerden en Werk, stichting Amsosa, UWV) | 🞎 | ____ keer |
| A5. | Fysiotherapeut | 🞎 | ____ keer |
| A6. | Diëtist | 🞎 | ____ keer |
| A7. | Psychiater, psycholoog of psychotherapeut **met eigen praktijk** | 🞎 | ____ keer |
| A8. | Psychiater, psycholoog of psychotherapeut in een **instelling** **voor geestelijke gezondheidszorg** | 🞎 | ____ keer |
| A9. | Psychiater, psycholoog of psychotherapeut op een **instelling voor verslavingszorg** | 🞎 | ____ keer |
| A10. | Psychiater, psycholoog of psychotherapeut op een **polikliniek** van een **psychiatrisch** **ziekenhuis** | 🞎 | ____ keer |
| A11. | Medisch specialist op een **polikliniek** van een **algemeen** **ziekenhuis** | 🞎 | ____ keer |
| A12. | Medisch specialist op een **polikliniek** van een **academisch** **ziekenhuis** | 🞎 | ____ keer |
| A13. | Pastorale en geestelijke verzorging | 🞎 | ____ keer |
| A14. | Thuiszorg: huishoudelijke hulp | 🞎 | ____ uur |
| A15. | Thuiszorg: persoonlijke verzorging | 🞎 | ____ uur |
| A16. | Wijkverpleegkundige | 🞎 | ____ uur |
| A17. | Iets anders, nl.: | 🞎 | ____ keer |

**B. Overige hulp**

De volgende vragen gaan over gebruik van overige voorzieningen en de hulp die u ontvangen heeft van bijzondere genezers, familie en vrienden in de **afgelopen 4 weken**.

|  | **Hoeveel uren heeft u de afgelopen 4 weken gebruik gemaakt van:** | **nee** | **Zo ja,**  **hoeveel uur?** |
| --- | --- | --- | --- |
| B1 | Hulp bij verwerken (bijv. patiëntenorganisaties; IPSO; Ingeborg Douwes Centrum; Stichting Les Vaux) | 🞎 | ____ uur |
| B2. | Zelfhulpgroep (bijv. AA; praatgroep van patiëntenvereniging) | 🞎 | ____ uur |
| B3. | Sportieve herstelprogramma’s (bijv. Herstel&Balans; Verder in Balans; Cytofys; Tegenkracht; Sportservice Noord-Holland; Nebas; Sportief Bewegen) | 🞎 | ____ uur |
| B4. | Hulp bij uiterlijke verzorging (bijv. Goed verzorgd, beter gevoel; Top Haarwerkers Gilde) | 🞎 | ____ uur |
| B5. | Bijzondere genezer / alternatieve geneeswijze (bijv. homeopaat; acupuncturist). | 🞎 | ____ uur |
| B6. | Hulp van familie of vrienden | 🞎 | ____ uur |
| B7. | Hulp van anderen, nl.: | 🞎 | ____ uur |
| B8. | Hulp van anderen, nl.: | 🞎 | ____ uur |

**C. Geneesmiddelengebruik**

Heeft u in de afgelopen **2 weken** de volgende medicijnen gebruikt?

|  | **Categorie**  Naam werkzame stof in medicijn  (commerciële naam) | **Ja (vul het aantal dagen / nachten in dat u het medicijn heeft gebruikt)** |
| --- | --- | --- |
| C1. | **Kalmerend, slaapbevorderend**  Bijv. alprazolam (xanax); chloordiazepoxide (librium); diazepam (valium); lorazepam (temesta); midazolam (dormicum); nitrazepam (mogadon); oxazepam (seresta); temazepam (normison); zolpidem (stilnoct); zopiclon (imovane); valdispert; clorazepinezuur (tranxene) | __ dagen / nachten |
| C2. | **Pijnstillend** (anders dan paracetamol, finimal, saridon of panadol)  Bijv. naproxen (aleve); diclofenac (voltaren); paracetamol-cordeïne; ibuprofen (advil, brufen, sarixell, nurofen); aspirine; fentanyl (durogesic); methadon; morfine (oramorph, MS contin, oxycontin, oxynorm); zaldiar; tramadol (tramal) | __ dagen / nachten |
| C3. | **Tegen gevoelens van neerslachtigheid of angst**  Bijv. amitriptyline (triptyzol); citalopram (cipramil); clomipramine (anafranil); duloxetine (cymbalta) ; escitalopram (lexapro) ; fluoxetine (prozac) ; fluvoxamine (fevarin); imipramine (tofranil); mirtazapine (remeron); moclobemide (aurorix); nortriptyline; paroxetine (seroxat); sertraline (zoloft); trazodon (trazolan); venlafaxine (efexor); fenelzine (nardil); tranylcypromine (parnate) | __ dagen / nachten |
| C4. | **Stoppen met roken**  Bijv. nicotinell, niQuitin, nicorette | __ dagen / nachten |
| C5. | **Voedingssupplementen, homeopathische geneesmiddelen, plantenextracten**  Bijv. vitaminen; mineralen; sporenelementen; visolie; aminozuren; St. Janskruid; Valeriaan; Melisse; producten van VSM; producten van A. Vogel | __ dagen / nachten |
| C6. | **Tegen taai slijm**  Bijv. acetyl cysteine; broomhexine; oral balance | __ dagen / nachten |
| C7. | Iets anders, nl.: | __ dagen / nachten |
| C8. | Iets anders, nl.: | __ dagen / nachten |
| C9. | Iets anders, nl.: | __ dagen / nachten |
| C10. | Iets anders, nl.: | __ dagen / nachten |

**Appendix I: CRA**

**Registratienr: Naam: Datum:**

Deze vragenlijst geeft inzicht in hoe u het geven van zorg aan uw partner ervaart, hoe dit invloed heeft op uw relaties met anderen, uw sociale activiteiten en dagelijkse bezigheden. Hieronder volgt een aantal uitspraken. Kunt u per uitspraak aangeven in hoeverre u het eens bent?

Het is de bedoeling dat u de afgelopen maand in ogenschouw neemt bij het invullen van de vragen.

|  | Heel erg mee oneens | Mee oneens | Eens noch oneens | Mee eens | Heel erg mee eens |
| --- | --- | --- | --- | --- | --- |
| Veel van mijn activiteiten zijn gericht op de zorg voor mijn partner |  |  |  |  |  |
| Ik ben gezond genoeg om voor mijn partner te kunnen zorgen. |  |  |  |  |  |
| Met het hele gezin zorgen we voor mijn partner. |  |  |  |  |  |
| Zorgen voor mijn partner is belangrijk voor mij. |  |  |  |  |  |
| Zorgen voor mijn partner kost al mijn energie. |  |  |  |  |  |
| Ik zorg graag voor mijn partner. |  |  |  |  |  |
| Ik moet dikwijls mijn werk of bezigheden onderbreken oom voor mijn partner te zorgen. |  |  |  |  |  |
| Mijn gezondheid is erop achteruit gegaan sinds ik voor mijn partner zorg. |  |  |  |  |  |
| Ik heb het gevoel dat mijn familie mij links laat liggen, sinds ik voor mijn partner zorg. |  |  |  |  |  |
| Zorgen voor mijn partner geeft me een goed gevoel. |  |  |  |  |  |
| Het is heel moeilijk hulp van de andere gezinsleden te krijgen bij het zorgen voor mijn partner. |  |  |  |  |  |
| Ik beschouw het als een voorrecht om voor mijn partner te zorgen. |  |  |  |  |  |
|  | Heel erg mee oneens | Mee oneens | Eens noch  oneens | Mee eens | Heel erg mee eens |
| Anderen laten de zorg voor mijn partner geheel op mij neerkomen. |  |  |  |  |  |
| Sinds ik voor mijn partner zorg, heb ik een aantal dingen moeten schrappen. |  |  |  |  |  |
| Ik heb er de pest over in dat ik voor mijn partner moet zorgen. |  |  |  |  |  |
| Door de voortdurende onderbrekingen is het moeilijk om tijd vrij te maken voor ontspanning. |  |  |  |  |  |
| Mijn familie (broers, zussen, kinderen) heeft de zorg voor mijn partner aan mij alleen overgelaten. |  |  |  |  |  |
| Sinds ik voor mijn partner zorg, lijkt het wel of ik altijd moe ben. |  |  |  |  |  |
| Ik wil graag voor mijn partner zorgen. |  |  |  |  |  |
| Sinds ik voor mijn partner zorg, ga ik minder bij familie en vrienden op bezoek. |  |  |  |  |  |
| Ik zal nooit in staat zijn voldoende zorg te geven om terug te doen wat mijn partner voor mij gedaan heeft. |  |  |  |  |  |
| De financiële middelen zijn toereikend. |  |  |  |  |  |
| Het is moeilijk om de zorg voor mijn partner te betalen. |  |  |  |  |  |
| Het zorgen voor mijn partner betekent een financiële belasting. |  |  |  |  |  |

**Appendix J: PTGI (versie VUmc**

**Naam: Datum:**

De diagnose kanker en de behandeling daarvan kan vervelende gevolgen hebben maar ook positieve, zowel bij de patiënt zelf als bij de partner. Met deze vragenlijst kunt u aangeven in hoeverre u positieve veranderingen heeft ervaren. Wilt u voor elke bewering hieronder de mate aangeven waarin deze verandering plaatsvond in uw leven als gevolg van de ziekte en behandeling van uw partner? U kunt dit doen door een kruisje te zetten bij het antwoord dat het beste bij u past.

|  | Niet  ervaren | Een heel klein beetje ervaren | Een klein beetje ervaren | Enigs-zins ervaren | In sterke mate ervaren | In zeer sterke mate ervaren |
| --- | --- | --- | --- | --- | --- | --- |
| Ik vind nu andere dingen belangrijk in het leven. |  |  |  |  |  |  |
| Mijn eigen leven heeft voor mij meer waarde gekregen. |  |  |  |  |  |  |
| Ik ontwikkelde belangstelling voor nieuwe dingen. |  |  |  |  |  |  |
| Ik heb meer zelfvertrouwen gekregen. |  |  |  |  |  |  |
| Ik heb meer inzicht gekregen in spirituele zaken. |  |  |  |  |  |  |
| Ik ben me er meer van bewust dat ik op mensen kan rekenen in moeilijke tijden. |  |  |  |  |  |  |
| Ik ben een nieuwe weg ingeslagen in mijn leven. |  |  |  |  |  |  |
| Ik voel me sterker met andere mensen verbonden. |  |  |  |  |  |  |
| Ik vind het nu makkelijker om mijn gevoelens te tonen. |  |  |  |  |  |  |
| Ik weet beter dan voorheen dat ik moeilijkheden aan kan. |  |  |  |  |  |  |
| Ik ben in staat meer met mijn leven te doen. |  |  |  |  |  |  |
| Ik ben meer dan voorheen in staat te accepteren dat dingen gaan zoals ze gaan. |  |  |  |  |  |  |
| Ik waardeer elke dag veel meer. |  |  |  |  |  |  |
| Er zijn nieuwe mogelijkheden beschikbaar, die er anders niet zouden zijn geweest. |  |  |  |  |  |  |
| Ik voel meer medeleven voor anderen. |  |  |  |  |  |  |
| Ik steek meer energie in mijn relaties. |  |  |  |  |  |  |
| Ik ben meer geneigd om dingen die verandering nodig hebben te veranderen. |  |  |  |  |  |  |
| Mijn religieuze geloof is sterker geworden. |  |  |  |  |  |  |
| Ik ben erachter gekomen dat ik sterker ben dan ik dacht. |  |  |  |  |  |  |
| Ik heb echt ervaren hoe geweldig mensen kunnen zijn. |  |  |  |  |  |  |
| Ik kan beter accepteren dat ik andere mensen nodig heb. |  |  |  |  |  |  |

**Appendix K**

VU medisch centrum

De Boelelaan 1117 postbus 7057 telefoon 020-444 4444

1081 HV Amsterdam 1007 MB Amsterdam fax 020-444 3688 www.VUmc.nl

Betreft: onderzoek naar de levensverhaalmethode

Amsterdam, december 2010

Geachte heer/mevrouw,

In het afgelopen jaar bent u behandeld voor kanker in het VU medisch centrum/ Nederlands Kanker Instituut/ Antoni van Leeuwenhoek ziekenhuis. Mogelijk hebt u ervaren dat kanker niet alleen lichamelijk, maar ook psychisch een ingrijpende ziekte is. Hoewel gebleken is dat psychische ondersteuning effectief is, wordt hier weinig gebruik van gemaakt. Ook het aanbod van psychische ondersteuning is nog gering.

Vanuit de afdeling Klinische Psychologie van de Vrije Universiteit Amsterdam, in samenwerking met het VU medisch centrum en het Nederlands Kanker Instituut (NKI), voeren wij momenteel een onderzoek uit naar de effecten van de levensverhaalmethode bij mensen met kanker. Wij hopen dat u wilt meewerken aan dit wetenschappelijk onderzoek. Met uw medewerking zijn wij beter in staat om de genoemde problemen in kaart te brengen en naar oplossingen te zoeken en de levensverhaal-methode verder te ontwikkelen. In deze folder zal uitgelegd worden wat de levensverhaalmethode inhoudt en wat deelname aan het onderzoek voor u betekent.

We adviseren u om voldoende tijd te nemen om deze informatie goed door te nemen en er goed over na te denken of u aan dit onderzoek wilt meewerken. Ook zult u er wellicht met anderen over willen praten. Hiervoor krijgt u uiteraard de gelegenheid. U kunt eventuele verdere vragen stellen aan de onderzoeker die u binnen een week contact met u op zal nemen en zal vragen of u mee wilt doen.

Met vriendelijke groet,

Gitta Kleijn, Prof dr. IM Verdonck-de leeuw,

Onderzoeker Hoogleraar *Leven met kanker*

**Depressieve klachten bij de ziekte kanker**

Voor veel mensen is de diagnose kanker een enorme schok. In welk stadium de ziekte ook is, het betekent onder andere angst voor aftakeling en de dood. Het besef een levensbedreigende ziekte te hebben kan leiden tot een crisis in het bestaan. Mensen kunnen het gevoel hebben zichzelf kwijt te raken in gevoelens van wanhoop, paniek en angst. Ieder verhaal is persoonlijk en dus anders, maar de klachten die voortkomen uit die psychische belasting vertonen veel overeenkomsten. Hieronder staan een aantal kenmerken van depressieve klachten:

- ‘minder lekker in je vel zitten’
- somberheid
- interesseverlies
- vragen over zingeving of waarom u kanker heeft gekregen
- gevoelens van hopeloosheid of waardeloosheid
- rusteloosheid
- prikkelbaarheid
- slaapproblemen
- veranderingen in eetlust en gewicht

Sommige van deze klachten gaan gepaard met de ziekte of de behandeling daarvan. Momenteel wordt er volop onderzoek gedaan naar het vroegtijdig signaleren van dergelijke klachten en het bieden van goede ondersteuning daarbij. In dit onderzoek willen we nagaan of de levensverhaalmethode daar een effectieve methode voor kan zijn.

**Levensverhaalmethode**

Wanneer mensen te horen krijgen dat ze kanker hebben stokt hun levensverhaal. Alle aandacht gaat naar de ziekte en het ziek zijn. Begrippen als verleden, heden en toekomst krijgen ineens een hele andere lading. Veel mensen hebben moeite de ziekte een plaats te geven en de lijn van het verhaal weer op te pakken. Vaak is het in zo’n stressvolle situatie lastig om, met name fijne en mooie herinneringen op te halen uit het verleden. Herinneringen die juist veel kracht kunnen geven in moeilijke tijden.

De dingen die we meemaken in ons leven slaan we op in ons zogeheten ‘’autobiografisch’’ geheugen. Dat is een deel van ons geheugen dat u kunt beschouwen als een archief van positieve en negatieve ervaringen. In dat geheugen liggen herinneringen vanaf de vroege kindertijd tot nu, ons hele levensverhaal. Veel herinneringen liggen onder het stof en lijken vergeten, tot er opeens iets gebeurt dat ons de ervaring doet herinneren. Herinneren maakt ons tot wie we zijn. De toegang tot dat autobiografisch geheugen is niet voor iedereen even makkelijk. Hierin spelen persoonlijkheid maar ook de huidige stemming een belangrijke rol. Ook is gebleken dat het autobiografisch geheugen trainbaar is. Met andere woorden: u kunt leren om gemakkelijker herinneringen op te halen.

Tijdens de levensverhaalmethode worden actief herinneringen opgehaald. Deze methode richt zich vooral op positieve, prettige gebeurtenissen en ervaringen in uw leven. In vier gesprekken van ongeveer één uur wordt door middel van een aantal vragen uw levensverhaal opgetekend.

Uit eerder onderzoek blijkt dat het praten over het levensverhaal helpt om ‘’alles op een rijtje’’ te krijgen en gebeurtenissen te verwerken. Mensen met een levensbedreigende ziekte zijn vaak op zoek naar nieuwe levensdoelen, maar zijn ook aan het terugkijken en herbeoordelen van bepaalde gebeurtenissen uit het verleden, kortom: het levensverhaal vorm te geven.

De levensverhaalmethode kan u helpen in het geven van betekenis aan bepaalde gebeurtenissen. Het vertellen van het levensverhaal kan er bijvoorbeeld voor zorgen dat mensen ‘lekkerder in hun vel komen te zitten’, of hoe dit formeel wordt genoemd: hun ‘kwaliteit van leven’ kan beter worden. . Dit gevolg willen wij graag verder onderzoeken. Ook willen wij de methode zo nodig aanpassen en verbeteren.

**Wat betekent deelname voor u**

Voor dit onderzoek willen wij twee groepen patiënten vergelijken: een groep die de levensverhaalmethode volgt en een groep die dat de methode niet volgt. Als u besluit mee te werken aan het onderzoek dan wordt u, op basis van toeval, ingedeeld in één van deze twee groepen. Of de onderzoeksgroep of een controlegroep. Bij de mensen in de onderzoeksgroep zullen de interviews afgenomen worden en er zullen er op drie verschillende momenten vragenlijsten worden afgenomen, Bij de mensen in de controlegroep zullen alleen de vragenlijsten afgenomen worden op dezelfde drie verschillende momenten.

Voor de levensverhaalmethode wordt u in totaal 6 keer thuis bezocht.

- 2 keer invullen van vragenlijsten (á 1 uur)
- 4 interviews over uw levensverhaal (á 1uur)

Een maand na het laatste bezoek zullen wij u voor de laatste maal verzoeken om vragenlijsten te beantwoorden. Via de post zult u vragenlijsten ontvangen, met een bijgevoegde retour envelop.

De interviewers die de gesprekken met u voeren zijn ervaren en opgeleid voor deze methode. De gesprekken worden u kosteloos aangeboden en vinden plaats op momenten die u het beste uitkomen. De gesprekken worden op een voice recorder opgenomen en als digitaal bestand aan u aangeboden na afronding van de gesprekken. De gegevens worden uitsluitend voor onderzoek gebruikt en vertrouwelijk behandeld. Dat wil zeggen dat uw naam nergens vermeld zal staan, maar dat de gegevens onder een code worden verwerkt.

Uw medewerking aan dit onderzoek is geheel vrijwillig. U bent op geen enkele manier verplicht om eraan mee te werken. U blijft de vrijheid houden om zonder opgaaf van redenen uw medewerking te staken. Als u besluit mee te werken aan het onderzoek dan zullen we u vragen een formulier te ondertekenen. Hiermee bevestigt u uw voornemen om aan het onderzoek mee te werken.

Zoals al het onderzoek in het VUmc is ook dit onderzoek voorgelegd aan de Medisch Ethische Toetsingscommissie (METc). Deze commissie beoordeelt of de onderzoekers voldoende zorgvuldig met de (gegevens van de) deelnemers omgaan. De METc van het VUmc heeft vrijstelling verleend van de verplichting om voor dit onderzoek een risicoverzekering af te sluiten omdat aan deelname geen risico’s verbonden zijn*.* Dat wil zeggen dat er geen nadelige effecten zijn als gevolg van deelname aan deze studie, voor zowel patiënten als hun naasten.

Indien u uw deelname aan het onderzoek wilt bespreken met een onafhankelijke arts, dan kunt u contact opnemen met prof. dr. P. Huijgens, afdeling Hematologie, telefoonnummer 020-4442604, of met uw huisarts.

Wij hopen dat u wilt meewerken aan dit wetenschappelijk onderzoek. Met uw medewerking zijn wij beter in staat om psychische gevolgen van kanker beter in kaart te brengen en passende hulp en ondersteuning aan te kunnen bieden. Mocht u na het lezen van deze brief nog nadere informatie willen dan kunt u altijd contact opnemen met de uitvoerder van het onderzoek, Gitta Kleijn, telefonisch te bereiken bij de afdeling Klinische Psychologie van de Vrije Universiteit Amsterdam (tel. 020-5988782) of via e-mail: [g.kleijn@psy.vu.nl](mailto:g.kleijn@psy.vu.nl).

Hoogachtend,

Gitta Kleijn

Hoofdonderzoeker

Mede namens

Prof. dr. C.R. Leemans, KNO-arts, hoofd-halschirurg

Prof. dr. P. Postmus, longarts

Prof dr. E.F. Smit, longarts

Dr. A. Becker, longarts

Prof. dr. I.M. Verdonk-de Leeuw, hoogleraar *Leven met kanker*

Prof.dr. P. Cuijpers, hoogleraar klinische psychologie

**Appendix L**

**Informed consent formulier - Respondentnummer:**

**Onderzoek: Effectiviteit van de levensverhaalmethode bij patiënten met kanker en hun partners.**

**TOESTEMMINGSFORMULIER (INFORMED CONSENT)**

Ik verklaar hierbij dat ik op voor mij duidelijke wijze, mondeling en schriftelijk, ben ingelicht over de aard, methode en doel, risico’s en belasting van het onderzoek. Mijn vragen zijn naar tevredenheid beantwoord.

Ik stem geheel vrijwillig in met deelname aan dit onderzoek. Ik behoud daarbij het recht deze instemming weer in te trekken zonder dat ik daarvoor een reden behoef op te geven.

Naam: ...............................................

Datum: ..........................................

Handtekening

………………………………………………………………………………………

Ik heb mondelinge en schriftelijke toelichting verstrekt over het onderzoek. Ik verklaar mij bereid nog opkomende vragen over het onderzoek naar vermogen te beantwoorden. Ik beloof geen persoonlijke informatie van deelnemer aan derden te verstrekken. De beslissing van de deelnemers om aan het onderzoek deel te nemen zal geen invloed hebben op de (na)zorg waarop iedere patiënt in dit ziekenhuis recht heeft.

Naam onderzoeker: ...............................................

Datum: ..........................................

Handtekening

………………………………………………………………………………………

**Appendix M**

VU medisch centrum

De Boelelaan 1117 postbus 7057 telefoon 020-444 4444

1081 HV Amsterdam 1007 MB Amsterdam fax 020-444 3688 www.VUmc.nl

Betreft: Informatiebrief voor partners

Amsterdam, december 2010

Geachte heer/mevrouw,

Met deze brief willen wij u informeren over het onderzoek naar kwaliteit van leven en de effecten van de levensverhaalmethode bij de partners van patiënten met kanker. Dit is een onderzoek wat uitgevoerd wordt gelijktijdig met en naast het onderzoek naar de effecten van de levensverhaalmethode bij mensen met kanker, waarover wij uw partner een informatiebrief hebben toegezonden. Indien uw partner besluit deel te nemen aan dat onderzoek, vragen wij ook om uw medewerking. Wat deelname aan het onderzoek voor u betekent, kunt u lezen in deze brief.

Er is (en wordt nog steeds) veel onderzoek gedaan naar de impact van de ziekte kanker op de persoon die er aan lijdt. Daaruit blijkt dat de ziekte, zowel lichamelijk als geestelijk, een grote invloed heeft op het leven van de patiënt. Veel minder duidelijk is wat voor impact de ziekte heeft op de partner van die persoon.

In dit onderzoek willen wij de kwaliteit van leven van de partner in kaart brengen, in relatie tot de belasting van de mantelzorg. Ook willen wij nagaan of er een effect te zien is van de levensverhaalmethode bij u als partner, ook al neemt u daar zelf niet direct aan deel. Om dit te kunnen onderzoeken vragen wij u om drie keer drie vragenlijsten in te vullen, op dezelfde momenten als de metingen bij uw partner. Deze vragenlijsten kunt u zelfstandig invullen. Het zal ongeveer 10 minuten per keer in beslag zal nemen. De gegevens zullen vertrouwelijk worden behandeld en anoniem worden bewaard.

Zoals al het onderzoek in het VUmc, is ook dit onderzoek voorgelegd aan de Medisch Ethische Toetsingscommissie (METc). Deze commissie beoordeelt of de onderzoekers voldoende zorgvuldig met de (gegevens van de) deelnemers omgaan. De METc van het VUmc heeft vrijstelling verleend van de verplichting om voor dit onderzoek een risicoverzekering af te sluiten omdat aan deelname geen risico’s verbonden zijn*.* Dat wil zeggen dat er geen nadelige effecten zijn als gevolg van deelname aan deze studie, voor zowel patiënten als hun naasten.

Uw medewerking aan dit onderzoek is geheel vrijwillig. U bent op geen enkele manier verplicht om eraan mee te werken. U blijft de vrijheid houden om zonder opgaaf van redenen uw medewerking te staken. Als u besluit mee te werken aan het onderzoek dan zullen we u vragen een formulier te ondertekenen. Hiermee bevestigt u uw voornemen om aan het onderzoek mee te werken.

Indien u uw deelname aan het onderzoek wilt bespreken met een onafhankelijke arts, dan kunt u contact opnemen met prof. dr. P. Huijgens, afdeling Hematologie, telefoonnummer 020-4442604, of met uw huisarts.

Wij hopen dat u deel wilt nemen aan dit onderzoek. Door uw medewerking kunnen wij de impact van de ziekte beter onderzoeken en de behoeftes aan ondersteuning bij partners in kaart brengen. Hierdoor kunnen de zorg en ondersteuning in de toekomst worden verbeterd. Mocht u na het lezen van deze brief nog nadere informatie willen, dan kunt u altijd contact opnemen met de uitvoerder van het onderzoek, Gitta Kleijn, telefonisch te bereiken bij de afdeling Klinische Psychologie van de Vrije Universiteit Amsterdam (tel. 020-5988782) of via e-mail: [g.kleijn@psy.vu.nl](mailto:g.kleijn@psy.vu.nl).

Met vriendelijke groet,

G. Kleijn

Hoofdonderzoeker

Prof dr IM Verdonck-de leeuw,

Psycholoog en hoogleraar *Leven met kanker*

| Afdeling hoofd/hals chirurgie  VU medisch centrum | Afdeling Longziekten  VU medisch centrum | Nederlands Kanker Instituut (NKI)  Antoni van Leeuwenhoekziekenhuis (AVL) | Vrije Universiteit Amsterdam  Faculteit der Psychologie en Pedagogiek |
| --- | --- | --- | --- |
| Prof.dr. C.R. Leemans, afdelingshoofd | Prof.dr.P.E. Postmus, afdelingshoofd | Dr. M.W.M. van den Brekel | Prof. dr. W.J.M.J. Cuijpers  Dr. J. Vos |
| Dr. R. de Bree | Prof. dr. E.F. Smit |  | Prof. dr. I.M.Verdonck – de Leeuw |
|  | Dr. A. Becker |  | Dr. B. Steunenberg |
|  |  |  | G. Kleijn |

**Appendix N:** Toestemming voor benadering

Code

(in te vullen door onderzoekers):

**TOESTEMMINGSVERKLARING**

*Wilt u één van de onderstaande mogelijkheden aankruisen?*

- Hierbij geef ik de onderzoekers van de *Vrije Universiteit* *Amsterdam* toestemming om mij te benaderen voor meer informatie en/of eventuele deelname aan het onderzoek ‘De Levensverhaalmethode’.
- Hierbij geef ik aan dat ik geen interesse heb in deelname aan het onderzoek ‘De Levensverhaalmethode’.

Naam :

Geboortedatum :

Adres :

Postcode en plaats :

Telefoonnummer :

E-mailadres :

Datum :

Handtekening :

**Appendix O verzekeringscertificaat**
